# Expanding the Spectrum of Diseases and Disease Associations Caused by *Edwardsiella tarda* and Related Species

**DOI:** 10.3390/microorganisms12051031

**Published:** 2024-05-20

**Authors:** J. Michael Janda, Muhammed Duman

**Affiliations:** 1 Kern County Public Health Laboratory, Bakersfield, CA 93306, USA; 2 Aquatic Animal Disease Department, Faculty of Veterinary Medicine, Bursa Uludag University, 16059 Bursa, Turkey; mduman@uludag.edu.tr

**Keywords:** *Edwardsiella*, *E. tarda*, edwardsiellosis, human infections, taxonomy, fish disease, edwardsiellosis in fish

## Abstract

The genus *Edwardsiella*, previously residing in the family Enterobacteriaceae and now a member of the family *Hafniaceae*, is currently composed of five species, although the taxonomy of this genus is still unsettled. The genus can primarily be divided into two pathogenic groups: *E. tarda* strains are responsible for almost all human infections, and two other species (*E. ictaluri*, *E. piscicida*) cause diseases in fish. Human infections predominate in subtropical habitats of the world and in specific geospatial regions with gastrointestinal disease, bloodborne infections, and wound infections, the most common clinical presentations in decreasing order. Gastroenteritis can present in many different forms and mimic other intestinal disturbances. Chronic gastroenteritis is not uncommon. Septicemia is primarily found in persons with comorbid conditions including malignancies and liver disease. Mortality rates range from 9% to 28%. Most human infections are linked to one of several risk factors associated with freshwater or marine environments such as seafood consumption. In contrast, edwardsiellosis in fish is caused by two other species, in particular *E. ictaluri*. Both *E. ictaluri* and *E. piscicida* can cause massive outbreaks of disease in aquaculture systems worldwide, including enteric septicemia in channel catfish and tilapia. Collectively, these species are increasingly being recognized as important pathogens in clinical and veterinary medicine. This article highlights and provides a current perspective on the taxonomy, microbiology, epidemiology, and pathogenicity of this increasingly important group.

## 1. Introduction

### 1.1. Historical Review

In the late 1950s and early 1960s, two national laboratories in Japan and the United States began working on a previously uncharacterized group of enteric-like bacteria [1]. Each set of strains shared common traits including elaboration of hydrogen sulfide (H_2_S) on TSI slants and indole production. Retrospective analysis of the data from these studies shows that each research group was working on the same microorganisms [2,3,4]. Both the National Institutes of Health (NIH, Japan) and the Center for Disease Control (CDC, USA) groups’ strains were unique among enterobacteria, did not resemble any previously described species, and exhibited similarity values < 50% when compared to named genera in the family *Enterobacteriaceae* [2]. Ewing and colleagues [5] proposed the name *Edwardsiella tarda* for this group in honor of Dr. P.R. Edwards of the CDC [6]. 

Whether *E. tarda* was pathogenic for humans was unknown. In 1964, King and Adler [3] reported the isolation of fecal strain CDC 40-1795-2 (later identified as *E. tarda*) from a hospitalized male with enteric fever and acute gastroenteritis. However, as early as 1966, reports began to surface concerning the possible pathogenicity of this species for humans when Gonzales and Ruffolo [7] described a post-trauma *E. tarda* wound infection in a six-year-old boy. Two years later, two additional publications (meningitis and septicemia) were published [8,9]. Table 1 lists some of the early seminal events in the description and characterization of the genus *Edwardsiella* and *E. tarda*.

### 1.2. Current Perspective

From its inception in the mid-1960s as a distinct enterobacterial genus and species, the interest and scientific importance of this taxon have continued to amplify over time. Using the search term “*Edwardsiella*” in PubMed^®^ [12], only five results (citations) were generated in 1980. However, these numbers have dramatically risen over the past few decades with 14 results in 2000, 73 results in 2010, and, finally, 153 results in 2020, the latter a more than 10-fold increase over 2000 figures. The clinical and medical importance of *E. tarda* in both scope and number of infections reported continues to increase globally [13,14]. Furthermore, as the genus has expanded in membership, species such as *E. ictaluri* and more recently *E. piscicida* have been recognized as causative agents of a variety of piscine diseases of economic importance, particularly in association with aquaculture [15,16]. The goal of this review is to provide an update on new and emerging trends linked to pathogenic groups within the genus *Edwardsiella*.

## 2. Edwardsiella Taxonomy

### 2.1. Nomenclature and Species Assignment

From 1965 to 1980, the genus *Edwardsiella* was represented by a single species, *E. tarda.* Studies conducted by Don Brenner and colleagues at the CDC in 1974 on 20 *E. tarda* strains using DNA-DNA hybridization (DDH) confirmed that this was a single tight DNA group that exhibited 82–96% interrelatedness among strains but only 20% to 29% relatedness to core members of the family such as *Escherichia coli* K-12 and *Salmonella* [17]. When the *Approved Lists of Bacterial Names* was published in 1980, the only species listed were *E. anguillimortifera* and *E. tarda*, both with the same type strain ATCC 15947 [18]. However, this list was short-lived because Grimont et al. [19] in 1980 described a second species of *Edwardsiella*, *E. hoshinae,* isolated from birds (*Fratercula artica*, *Phoenicopterus ruber*), reptiles (*Varanus* spp.), and water. This was quickly followed by the description of *E. ictaluri*, the causative agent of enteric septicemia in catfish [20].

For more than three decades, the number of species in the genus *Edwardsiella* remained stable at three (*E. tarda*, *E. hoshinae*, *E. ictaluri*). Subsequent phylogenetic investigations of *E. tarda* fish isolates revealed that these strains did not belong to *E. tarda sensu stricto* but rather represented two new species, *E. piscicida* [21] and *E. anguillarum* [22]. Subsequent studies have confirmed previous findings that most pathogenic *E. tarda* fish strains have been misidentified. An investigation of 49 fish isolates of *E. tarda* subjected to AFLP (amplified fragment length polymorphism), MLSA (multilocus sequence typing), and in silico DDH revealed 46 (94%) to be *E. piscicida* and 2 (4%) to be *E. anguillarum* [23].

Current members of the genus *Edwardsiella* are listed in Table 2.

### 2.2. Classification: Past to Present

When the genus *Edwardsiella* was first created in 1965 [5], it was placed in the family *Enterobacteriaceae* under a new tribe, the Edwardsiellae. The basis for this proposal was largely phenotypic in nature in that it shared many family-specific traits with other members of the enterobacteria being Gram-negative, oxidase-negative, peritrichously flagellated, containing fermented glucose, and nitrate reductase-positive. In addition, although less consequential, it was isolated frequently from similar anatomic sites as other groups, such as the gastrointestinal tract. Finally, the fact that the “Asakusa” and “Bartholemew” groups, thought to be identical to CDC 1483-59, had no standing in nomenclature helped precipitate the taxonomic proposal to unify global communication and research on this cluster.

The tribe concept for the enterobacteria dissipated in the mid-1980s as more genera and species were assigned to this family. Even though both human and reptile strains of *E. tarda* produced a homogenous group by DDH, this taxon was only distantly related to core members (e.g., *E. coli*, *Shigella*, *Enterobacter*, *Salmonella*) of the family based upon 20% to 29% reassociation kinetics [17]. In the mid-1980s, the CDC grouped all genera in this family into one of three groups (distantly related, moderately related, or closely related) based on DNA relatedness [25]. Further phylogenetic investigations assessing various housekeeping genes and other markers almost invariably placed this genus at the extreme periphery of dendritic trees of the family *Enterobacteriaceae*, similar to that observed for other such groups as *Plesiomonas* and the tribe *Proteeae.*

Two pioneering studies published by Radley S. Gupta and coauthors in 2016 and 2017 [26,27] helped to clarify the exact phylogenetic and taxonomic positions of the current taxa in the family *Enterobacteriaceae*. Phylogenomic studies employing 78 genome-sequenced enterobacterial species based upon 2487 core genome proteins and 118 other conserved proteins identified six main clades in the family [26]. This was further supported by the analysis of average amino acid identity and 16S rRNA sequence similarity. The study further identified 88 conserved signature indels uniquely shared by specific members of the family. Based upon this and additional phylogenetic data, the proposal was made to reclassify many members of the family *Enterobacteriaceae* into six new families within the order *Enterobacteriales* [27]. This reclassification has been widely accepted. Currently, the genus *Edwardsiella* resides in the family *Hafniaceae* along with *Hafnia* and *Obesumbacterium* [27,28].

### 2.3. Taxonomic Issues

There are several outstanding taxonomic issues regarding the genus *Edwardsiella*. Presently, two named species (*E. tarda* and *E. anguillimortifera*) exist with the same type strain (ATCC 15947). Both taxa were listed on the Approved List of 1980 [18] and were validly published [24]. Although the biochemical properties listed for both species are identical in primary characteristics, the sources of strains for both species were different, and no nomenclature type strain was identified or deposited when the species name ‘*Paracolobactrum anguillimortiferum*’ was originally proposed by Hoshina in 1962. Furthermore, no extant strain of *P. anguillimortiferum* appears to exist. According to Tindall [29], these two groups are not homotypic but heterotypic synonyms. Based upon various rules of the Code, the name *E. anguillimortifera* is illegitimate and may not be used.

A second more intriguing issue is the phylogenetic depth and complexity of the genus. Recent MLST studies employing 10 housekeeping genes support the delineation of five distinct species in the genus *Edwardsiella*, namely *E. tarda*, *E. hoshinae*, *E. ictaluri*, *E. piscicida*, and *E. anguillarum* [30]. The complexity of the genus in terms of species is unknown. Recent phylogenetic reclassifications of “typical” and “atypical fish pathogenic *E. tarda* strains” into two new species (*E. piscicida* and *E. anguillarum*) expand this complexity [22,31]. These investigations also indirectly bring into question the biochemical diversity, true geographic range and distribution, and global disease spectrum associated with *E. tarda sensu stricto*. Outlier strains from such surveys as NCIMB 2034 [21] that do not comfortably fit into any defined taxon may additionally reflect a more diverse genetic population [22].

## 3. Epidemiology

### 3.1. Environmental Distribution—Overview

Unfortunately, no extensive surveys on the natural habitat(s) of *Edwardsiella* species have ever been undertaken. According to Austin and Austin [32], *Edwardsiella* has been reported from 25 countries in Europe, Asia, Australia, Africa, North and Central America, and the Middle East. Currently, *Edwardsiella* has been reported in a broad geographical range, including Asia, the USA, and Europe, and has been found in more than 25 fish host species. This indicates that *Edwardsiella*, which is typically found in freshwater and marine environments, has pathogenic potential for animals inhabiting these ecological niches [33]. *E. tarda* has been adapted to live in diverse environmental conditions and can be isolated from a wide range of water salinity (0–4% NaCl), pH (4.0–10), and temperatures (14 to 45 °C) [1,33,34], explaining to some extent its capability to cause disease not only to freshwater and marine fish but also to terrestrial animals. Leung et al. [33] proposed that *Edwardsiella* primarily inhabits three niches. The first is aquatic environments, where free-living or communal bacteria are exposed to stresses such as changes in temperature, salinity, and nutrient availability. Antibiotic-contaminated environments are a concern in aquaculture farms, where bacteria in fish hosts may encounter sub-inhibitory or inhibitory antibiotic concentrations. Pathogenic bacteria use virulent genes to attach, invade, colonize host cells, and spread within the host’s body, causing systemic infections [33].

Two additional ecological groups of hosts, cold-blooded animals and fish, could be considered reservoirs of *Edwardsiella* species. While human infection caused by *E. tarda* is rare, there is valuable information in the literature from individual case reports on risk factors associated with Edwardsiellosis [14]. Since Sakazaki and Murata [35] first suggested *E. tarda* as a normal intestinal inhabitant of snakes, it has been recognized that a wide range of reptiles and amphibians, including snakes, crocodiles, alligators, toads, lizards, frogs, and turtles, are possible natural reservoirs for *E. tarda* [36]. On the other hand, Van Damme and Vandepitte [37] reported the isolation of *E. tarda* from various kinds of river fish in Zaire. It is considered that river fish and their environment seem to constitute the natural habitat of *E. tarda* and to be the most probable source of human infection, at least in tropical countries. *E. tarda* has been infrequently isolated from warm-blooded animals such as dogs, pigs, cattle, monkeys, rats, panthers, skunks, seals, sea lions, and birds [1]. 

More specifically, as noted in Abbott and Janda [1], freshwater fish appear to be the primary habitat for at least two *Edwardsiella* species, *E. tarda* and *E. ictaluri*. *E. ictaluri* is almost exclusively associated with ictalurid fish, although this species has occasionally been cultured from non-ictalurid fishes [38]. *E. tarda* has a broader host range among piscine species [1]. Other marine life from which *E. tarda* can occasionally be isolated includes mullet (*Mugil cephalus*), cultured sea bream (*Evynnis japonicus*), seals, sea lions, mussels, and clams [1,28]. Both species are also recovered from environmental samples associated with aquatic ecosystems, including pond water and sediment samples [39]. The *E. hoshinae* species is phenotypically and genotypically distinct from other *Edwardsiella* spp. [19]. It is most often recovered from avian and reptilian hosts and is not known to cause disease in humans, birds, reptiles, or fish [40]. One of the other isolated and identified species, *E. piscicida*, which was previously reported as *E. tarda*, has been reported from European eel (*Anguilla anguilla*), turbot (*Scophthalmus maximus*), Korean catfish (*Silurus asotus*), marbled eel (*Anguilla marmorata*), and Japanese eel (*Anguilla japonica*) from 1989 to 2009 in Norway, Southern Europe, Northern Europe, United Kingdom, China, and South Korea [21]. Another novel species is *E. anguillarum* based on polyphasic phenotypic and genomic reclassification of *Edwardsiella* phylogroup isolates recovered from diseased eels, which had previously been classified as *E. tarda* or the newly established *E. piscicida* [22]. Whole-genome sequencing that was conducted on the strain EA011113, isolated from farmed *Diplodus puntazzo* after an edwardsiellosis outbreak in Greece, confirmed it as a new clinical strain of *E. anguillarum* [41]. 

### 3.2. Factors Regulating Environmental Distribution

Few reports describe the free-living aspects of *Edwardsiella* species, such as *E. tarda*, *E. piscicida*, and *E. anguillarum*, in aquatic environments. Many important questions remain unanswered about the organism’s adaptation to aquatic environments, as well as the major differences between free-living isolates and those isolated from diseased hosts.

The genus *Edwardsiella* has evolved various mechanisms to cope with rapid environmental changes occurring naturally or due to anthropogenic factors in aquatic environments. Naturally occurring events encompass alterations in salinity, pH, nutrient levels, temperature, and other physical parameters. Research indicates that *Edwardsiella* species, such as *E. piscicida* and *E. ictaluri*, utilize diverse mechanisms to combat various stresses in aquatic environments [16,33,42]. These bacteria employ internal molecular “tools” and may acquire additional foreign genes or gene clusters from the aquatic microbiome to ensure their survival. In recent studies, authors have documented the genomic characteristics of *Edwardsiella* species to adapt to salinity and pH shifts, temperature changes, and nutrient starvation in addition to biofilm formation in stressful conditions [33,43]. For example, the *E. tarda* strain likely evolved its virulence and adaptation to a broad range of hosts using Type III and VI secretion systems (T3SS and T6SS) and iron scavenging-related genes. These systems and genes are key evolutionary factors that facilitated the strain’s evolution [43]. Hence, the *Edwardsiella* species can easily transfer to different environments by being a natural inhabitant of aquatic environments. 

### 3.3. Human Infections

Edwardsiellae are not considered to be normal microbiota of the human microbiome, including the gastrointestinal tract [44]. The limited data suggest that the clinical frequency of the fecal carriage rate of *E. tarda* ranges between 0% and 0.8% [44]. This implies that almost 100% of all *Edwardsiella* infections are acquired from exogenous sources. Various factors regulate such infections including the type of reservoir-to-person transmission, strain virulence, infectious dose, and risk factors or comorbid conditions associated with the infected host. 

**Figure 1:**
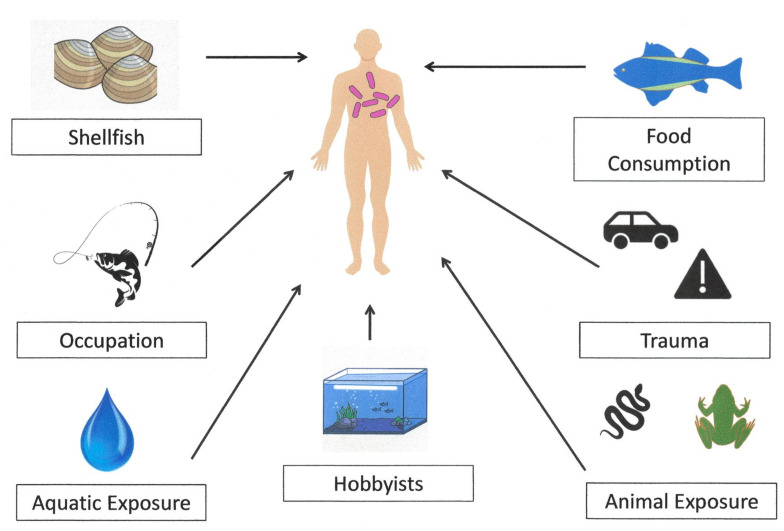
Graphic representation of potential sources of environmental exposure to human *Edwardsiella* strains.

Figure 1 illustrates some of the primary routes by which *E. tarda* can be transmitted to humans resulting in infection. These routes can be broken down into five general areas, namely (1) ingestion of contaminated consumable products or foods naturally harboring edwardsiellae (fish, shellfish), (2) exposure or direct contact with contaminated aqua systems (freshwater, marine environment), (3) trauma precipitating a penetrating injury, (4) animal or zoonotic exposure (handling, trauma), and (5) occupational exposure. With minor exceptions (car accidents, glass), there is considerable overlap in many of these categories with a common underlying theme of water [45].

*Edwardsiella* infections have been reported from many different geographical locales. A comprehensive review by Hirai and others [46] of 46 publications involving *E. tarda* bacteremia identified Japan (45.5%), the United States (US) (15.6%), and the Republic of China (Taiwan) (13%) as having the most illnesses, although no cases in the southern hemisphere were identified in this analysis. The high incidence of *E. tarda* sepsis from Asia/SE Asia is probably a reflection of their dietary customs (fish, eels). In the US, most infections (66.7%) were centered around the Gulf of Mexico or other coastal states [46]. Some studies on *E. tarda* infections (sepsis, wounds) suggest that there is a seasonal variation in *E. tarda* disease (May–November) [45,46], although this is not a universal finding [47]. Slaven et al. [45] and Healey et al. [48] postulate that this may be due to increasing concentrations of edwardsiellae in rising water temperatures and an elevated risk of exposure to contaminated vehicles due to heightened recreational activities. More definitive data are needed from systematic surveys of gastroenteritis-associated illnesses over a protracted period.

#### 3.3.1. Vehicles of Infection

Several vehicles have been associated with *E. tarda* infections. Unfortunately, most of these epidemiologic associations have been retrospective or anecdotal in nature once the genus and species of the pathogen were identified. Table 3 lists a number of proposed sources of infection reported in various publications. In three instances, a direct link between an environmental source and a clinical isolate has been reported. Years ago, a case of *E. tarda* enteritis was connected to a tropical aquarium fish isolate producing the exact same API 20E profile [49]. In 2006, a liver abscess infection in a 26-year-old male was linked to an isolate recovered from a village pond’s water where the patient had repeatedly bathed [50]. Both isolates generated an unusual *E. tarda* biotype, biotype 1 [50]. Finally, *E. tarda* was recovered from a stool sample of a caregiver who failed to wash their hands prior to a peritoneal bag exchange, resulting in an episode of *E. tarda* peritonitis [51]. 

##### Food Consumption

The most common vector involved in E. *tarda* disease transmission is the consumption of contaminated food resulting in gastroenteritis. Frequent foods linked not only to gastroenteritis but also sepsis include fish, grilled eel [52,53], and oysters [48]. The ingestion of raw fish leading to a variety of *E. tarda* gastrointestinal complications (enteritis, colitis, food poisoning) has taken many different forms. This includes prepared dishes such as ceviche, sashimi, and sushi [47,53]. Other less frequently encountered food sources of infection are listed in Table 3.

##### Animal-Associated Trauma

Even though *E. tarda* has frequently been recovered from cold-blooded animals including reptiles, lizards, and amphibia, there is virtually no evidence to support a documented role for any of these groups in infective processes. The most common *E. tarda* syndrome associated with animal-associated trauma is wound infections (cellulitis, myonecrosis) resulting from penetrating injuries to the extremities. The most common injury observed involves catfish punctures, stings, or bites [54]. Other complications include fishbone pricks or punctures resulting in wound infections or sepsis [45,54].

On two occasions, turtles have been implicated in human infections. Nagel and associates [55] described a case of gastroenteritis in a 37-year-old female who inadvertently took a drink from a glass her son had used when cleaning a turtle tank. A second possible case with limited information concerns *E. tarda* sepsis involving environmental exposure to a turtle [47]. Over the past 15 years, at least four cases of *E. tarda* infection have been associated with goldfish and related ecosystems (Table 4). Two of these infections (gastroenteritis and urinary tract infections) involved pediatric patients, and two others involved a young adult and an elderly male. Both adult patients were septic with *E. tarda*. While most of the clinical findings varied considerably in these cases, a common underlying theme was the handling, taking care of, or playing with goldfish in a tank or aquarium. The case report by Gilani and others [56] indirectly suggests that the dead goldfish may have also been infected with edwardsiellae. 

##### Aquatic and Occupational Exposures

Most aquatic-related *E. tarda* infections involve indirect rather than direct exposure to water itself. Exceptions to this rule include near-drowning events [59], repeatedly bathing in a village pond [50], or exposure to *E. tarda* in freshwater lakes. However, in most instances of aquatic exposure, water is the medium rather than the direct source of infection. An example is the case of a 48-year-old man who fell into brackish water and lacerated his forearm on a submerged brick [45]. Whether the water or brick was the source of exposure is unknown. Unusual aquatic exposures leading to infection or colonization have been described and include a mother who participated in a baptism by immersion in a lake and another mother who washed her clothes in a river [46].

Because of the direct or indirect association of *E. tarda* infections with the aquatic environment, occupations or professions associated with these ecosystems (freshwater, marine) are uniquely susceptible to infection. The most common occupations reportedly involved in *E. tarda* infections include fishing/fisherman and fishmongers [45,46,60]. Although not documented, the fact that many vertebrate animals, including reptiles, can be colonized with edwardsiellae suggests that veterinarians, zookeepers, and pet store employees can be at risk as well.

#### 3.3.2. Risk Factors

While the vast majority of *Edwardsiella* gastrointestinal tract and wound infections occur in healthy individuals or in persons with only minor abnormalities, the opposite situation is true for those presenting with systemic *E. tarda* disease, which includes sepsis and hepatobiliary illnesses. Extraintestinal disease most often occurs in persons with various comorbid conditions, the principal ones being malignancy and liver dysfunction. While chronic alcoholism can lead to predisposing conditions of infection like Laennec’s cirrhosis, the disorder itself of chronic ethanol abuse has precipitated cases of pneumonia [59], a gastric submucosal abscess [61], and fulminant necrotizing fasciitis [62]. Patients warned of excess alcohol consumption have ignored physicians’ advice that preceded their fatal episodes of systemic *E. tarda* infection [62]. Hepatitis C has also been reported as an underlying complication associated with a variety of extraintestinal infections including bacteremia, myonecrosis, and pelvic inflammatory disease [54]. Other less prominent predisposing risk factors sometimes linked to *E. tarda* infection include diabetes mellitus, non-alcoholic steatohepatitis, and gastrectomy [46,48,53,54]. 

### 3.4. Zoonotic Infections and Distribution

While the term zoonotic diseases remained relatively unknown in many countries, the COVID-19 pandemic that swept the world in December 2019, leading to the deaths of over 7 million people globally (according to WHO reports), has starkly highlighted the significance of zoonotic infections worldwide. No one can guess which zoonotic agent will turn into a pandemic because bacteria and viruses have plasticity for virulence characteristics; thus, they can cause severe mortality when they find suitable conditions such as environment, temperature, or host. Among these zoonoses, *Edwardsiella* behaves like an underhand zoonotic pathogen and poses a significant risk when considering the more than 100 public aquariums and over the 100 million home aquariums kept in fish, reptiles, or turtles [63]. 

The order *Enterobacteriales* includes the primitive genus *Edwardsiella* [1,28,32], which comprises six species: *E. anguillimortifera*, *E. tarda*, *E. hoshinae*, *E. ictaluri*, *E. piscicida*, and *E. anguillarum* [28,30,44] (see Table 2). A review of the currently published literature review indicates there are no reports on *E. anguillimortifera*, *E. piscicida*, and *E. anguillarum* isolation from human specimens. After the initial report of *E. hoshinae* by Grimont et al. [19], the distribution of the bacterium in the environment and hosts has remained a mystery. There are only a few reports on the isolation of *E. hoshinae* from ducks, pigs [64], human feces [65], puffins, lizards, water, and fish in Europe, Asia, the USA, and East Africa regions [66]. Despite the reports from both human feces and animals, the transmission routes and zoonotic importance remain unknown. 

Fishborne zoonotic cases of *E. tarda* infection are based upon reports from 40 years ago regarding a two-month-old Belgian infant where *E. tarda* was isolated from tropical aquarium fish at the same time [49]. *E. tarda* commonly inhabits the intestinal flora of aquatic animals and has been reported from over 25 fish species, but it can also cause intestinal and extraintestinal infections in reptiles, amphibians, birds, and mammals, including humans, by unknown mechanisms [67,68,69]. In addition, *E. tarda* has been isolated from invertebrates [70], cows, pigs, dogs, and Weddell seals [5,71,72]. A wide geographic distribution and host range of *E. tarda* is evident from these reports. *E. tarda* is becoming an important pathogen for global public health due to the increasing contact between aquatic animals and humans. *E. tarda* was first described by Ewing et al. in 1965 [5], and while rarely encountered in humans, clinical cases have increased in recent decades. The most common manifestation of edwardsiellosis infection in humans is gastroenteritis, which is more prevalent in tropical and subtropical climates [11,14,28,71]. The medical history of persons infected with *E. tarda*, especially those suffering from skin and gastrointestinal infections, often reveals exposure to freshwater environments or activities (swimming, diving, and fishing). Nucci et al. [73] have suggested that human stools could have contaminated freshwater from which fish had been caught containing *E. tarda* agents. The proof of zoonotic transmission of *E. tarda* is the isolation together with *E. coli* from fish harvested from Ethiopia for human consumption [74]. 

According to the definition by Leung et al. [33], “*Edwardsiella*, a water-living bacterium that adapts to free- and host-living lifestyles”, the members of the genus have an important risk assessment where water and humans come in contact.

#### 3.4.1. Piscine Species

Although *E. tarda* is commonly linked to human infections, *E. ictaluri*, *E. piscicida*, and *E. anguillarum* were derived from fish sources like their own names, *ictalurid*, piscine, and anguilliform fish species, respectively [20,21,22]. *E. anguillimortifera* and *E. hoshinae* were differentiated from *E. tarda*, and there were no reports from fish sources [19,29]. 

*E. ictaluri* is almost exclusively associated with ictalurid fish such as Channel catfish (*Ictalurus punctatus*) and brown bullhead (*Ictalurus nebulosus*), although this species has occasionally been cultured from non-*ictaluri*d fishes; up to 44 fish species in four continents are known to be susceptible such as Rhamphichthyid green knife fish (*Eigenmannia virescens*), Cyprinad danio (*Danio devario*), and ornamental fish (*Puntius conchonius*) [1]. *E. ictaluri* was also found in moribund zebrafish held in quarantine at two universities in two US states. Edwardsiellosis is a severe systemic disease in zebrafish that is characterized by tissue necrosis and the presence of large numbers of extracellular and intracellular bacteria, often within macrophages [75]. In total, seven families of catfish have been found to be susceptible to *E. ictaluri*, including *İctaluridae*, *Bagridae*, *Clariidae*, *Pangasiidae*, *Ariidae*, *Siluridae*, and *Plotosidae*. Fish other than catfish have been reported to have susceptibility in 10 families, including *Plecoglossidae*, *Sternopygidae*, *Cyprinidae*, *Cichlidae*, *Salmonidae*, *Moronidae*, *Anguillidae*, *Percichthyidae*, *Balaenopteridae,* and *Pleuronectidae* [15].

*E. tarda* is widely recognized as one of the leading pathogens of freshwater and marine-farmed fish throughout the world. *E. tarda* has a broad host range among piscine species, infecting more than 20 commercially important fish species, including rainbow trout (*Oncorhynchus mykiss*), chinook salmon (*Oncorhynchus tshawytscha*), tilapia (*Oreochromis* sp., Nile tilapia (*Oreochromis niloticus*), channel catfish (*Ictalurus punctatus*), largemouth bass (*Micopterus salmoides*), eel (*Anguilla anguilla*), mullet (*Mugilidae*), barramundi (*Lates calcarifer*), sea bream (*Sparus aureta*), striped bass (*Dicentrarchus* sp.), yellowtail (*Seriola lalandi*), common carp (*Cyprinus carpio*), freshwater catfish (*Ictalurus punctatus*), angel fish (*Pterophyllum* sp.), Japanese flounder (*Paralichthys olivaceus*), turbot (*Scophthalmus maximus*), koi (*Cyprinus* sp.), oyster toadfish (*Opsanus tau*), Oscar fish (*Astronotus ocellatus*), and pacu fish (*Piaractus mesopotamicus*) [65,76,77]. As we mentioned, *E. tarda* is a bacterium that water and, thus, fish acquire through cross-continental movement, especially in countries where aquaculture facilities are high in activity. For example, common reports stem from the USA, Europe, and Asia where catfish, salmonids, and carp species are cultured in a high density [45,67,69,73,77]. *E. tarda* has been reported in various countries, including Egypt, Ethiopia, Indonesia, Taiwan, Bangladesh, Venezuela, Uganda, Thailand, and Nigeria. These countries have tropical climates and are home to both local fish species and commonly cultured fish [74,78,79,80,81,82,83,84,85].

The growth characteristics and biochemical and genetic differences suggest that the isolates from fish previously identified as *E. tarda* were misclassified. Therefore, Abayneh et al. [21] classified a novel species within the genus *Edwardsiella* and proposed the name *Edwardsiella piscicida*. In this first description, *E. piscicida* was originally defined from European eel (*Anguilla Anguilla*), turbot (*Scophthalmus maximus*), Korean catfish (*Silurus asotus*), marbled eel (*Anguilla marmorata*), and Japanese eel (*Anguilla japonica*) in Norway, Finland, Southern Europe, Scotland (UK), China, and South Korea [21,23,86,87]. After the reclassification and identification of *E. piscicida*, the isolate was also isolated from ayu, barramundi, seabream, blotched fantail stingray, Japanese flounder, koi, rainbow trout, serpae tetra, sole, and whitefish more than ten different countries [23]. A more recent report showed that *E. piscicida* is an important pathogen for fish species that caused mass mortality of over 65% of marbled eel in Korea [88].

Like *E. piscicida*, *E. anguillarum* has also resulted from a reclassification within the genus *Edwardsiella*, which was previously identified as *E. tarda* [22]. The first study to describe *E. anguillarum* reported its presence in Japanese eel, marble eel, European eel, and seabream in China and Japan [22]. Due to its isolation from eels, the bacterium was named *E. anguillarum*, but it has since been reported as a pathogenic species in catfish, Nile tilapia, cultured sharpsnout seabream, and zebrafish in the USA and Greece. Wang et al. [89] showed that *E. anguillarum* is a highly pathogenic species for American eels (*Anguilla rostrata*), with acute mortality rates of up to 76.4%; however, there are no reports of *E. anguillarum* from a wide range of countries where farmed fish production is high.

#### 3.4.2. Vertebrate Species

Edwardsiellosis outbreaks represent significant epidemiological events when these bacteria affect multiple vertebrate species sharing the same aquatic environment. Although the epidemiological significance of the presence of *Edwardsiella* species in vertebrates requires further investigation, some reports indicate a high likelihood of transmission from aquatic environments to aquatic vertebrates, such as seals and sea lions [28,44,90,91]. Aquatic mammals have also been reported as carrier or incidental hosts for *E. tarda* [44]. 

#### 3.4.3. Reptiles

Reports on *Edwardsiella* infections or the presence of the species in animals, excluding fish, are commonly based on data from 30 years ago. Roggendorf and Müeller [92] isolated *E. tarda* from 20% of turtles, 12% of snakes, and 3% of lizard specimens, suggesting that turtles may harbor this organism. In a study of wildlife in Panama, *E. tarda* was found in 6% of toads and 5% of snakes examined [93]. However, it is generally considered part of the gastrointestinal microbiota of crocodilians [94,95,96], although recent evidence has shown that this bacterium can cause fatal septicemia in farmed hatchlings [97,98]. Rehman et al. [98] have recently supported the idea that *Edwardsiella* infections in crocodiles can lead to septicemia among hatchlings during the winter and spring seasons, with *E. tarda* as a primary pathogen. In another instance, *E. tarda* was cultivated as a pathogenic bacterium in grass snakes, causing multiple subcutaneous nodules [99]. The habitat of *E. hoshinae* other than fish is not well known. *E. hoshinae* is also found in the bacterial microbiota of reptiles and avians, and no infectious diseases were caused by this species [64,66]. In the only publication on this species, *E. hoshinae* was isolated from lizards [19].

#### 3.4.4. Miscellaneous Groups (Birds, etc.)

*Edwardsiella* has been associated with disease in several vertebrate species, but its pathogenic role in warm-blooded animals is not known. *E. tarda* has been isolated from warm-blooded animals, including dogs, pigs, cows, monkeys, rats, panthers, skunks, seals (*Arctocephalus gazella*, *Leptonychotes weddelli*), sea lions, and birds. 

One other important epidemiological characteristic of *Edwardsiella* is its adaptive ability because the species can adapt to aquatic birds (*Stercorarius maccormicki*, *Larus dominicanus*) and also birds/animals (*Macromectes giganteus*, *Stercorarius lonnbergi*, *Pygoscelis adeline Arctocephalus gazella*) in Antarctic environments [100]. Although outbreaks involving domestic ducks and wild birds are not common in the literature, *E. tarda* has been identified as the cause of acute septicemia in ducks (*Anas platyrhynchos domesticus*) and egrets (*Egretta thula*). Supporting *Edwardsiella* transmission to aquatic birds, *E. tarda* has been associated with acute enteritis in aquatic bird species, including adult ducks and egrets [101]. Authors have reported *E. tarda* from different bird species such as bald eagles, blue herons, brown pelicans, gulls, king vultures, loons, ostriches, penguins, and sandhill cranes over 20 years ago [46,55,64,70,101]. To support this evidence, there is a large survey on the prevalence of *E. tarda* in Antarctic wildlife in bird species, including southern giant petrels, brown skuas, south polar skuas, kelp gulls, greater sheathbills, Adelie penguins, Adelie penguin eggs, gentoo penguins, chinstrap penguins, and Weddell seals [100]. The literature clearly shows that *Edwardsiella* can survive extreme conditions, as it was found in Antarctica in native aquatic bird species. Other marine life from which *E. tarda* can occasionally be isolated includes mussels, clams, bullfrogs, and ducks [44,90,91]. In the only publication on this species, *E. hoshinae* was isolated from two puffins, a flamingo, and water [19].

## 4. Diagnostic Microbiology

### 4.1. Genus Characteristics

From its preliminary description in 1965 as a single-species genus until recent phylogenetic investigations, *Edwardsiella* has resided in the family *Enterobacteriaceae* for more than 50 years [5]. The primary reasons for this original proposal and classification included (1) possessing basic phenotypic features common with other members of the family at that time, (2) strains in the CDC collection primarily originating from the gastrointestinal tract of humans (74%), which was similar to other enteric genera such as *Salmonella*, *Shigella*, and *Escherichia*, and (3) the need to provide taxonomic resolution (standing in the literature) for the “bacterium 1483-59” group to avoid the usage of a number of different vernacular designations. Some of these “common” phenotypic features included Gram-negative bacilli, oxidase-negative status, the occurrence of D-glucose fermentation, and the reduction of nitrate to nitrite.

As the family expanded in terms of absolute numbers of genera and species in the 1970s and early 1980s, a slightly expanded list of family-associated biochemical characteristics was used for inclusion in this family in addition to DDH [102]. These characteristics are listed in Table 5 [1,14,103]. A defining feature of these traits was the possession of the enterobacterial common antigen, or ECA [103]. *Edwardsiella* possessed all of the features listed in Table 5 with the singular exception of the inability to ferment D-xylose.

Several other properties help to define this genus. These features include motility by means of peritrichous flagellation, resistance to colistin but susceptibility to penicillin, and NAD and amino acid requirements for growth [1]. A cardinal feature of this genus, excluding *E. hoshinae*, is its inability to ferment most sugars except for D-glucose and maltose. The term “*tarda*” means slow but actually refers to the inactivity of this species against most carbohydrates including lactose, D-mannitol, and sucrose.

### 4.2. E. tarda

*E. tarda* is the only *Edwardsiella* species known at present to be pathogenic for humans. On the basis of population and evolutionary analyses, genetic studies, and phylogenetic investigations, it is clear that many previously reported “*E. tarda*” isolates recovered from environmental sources and fish are not, in fact, *E. tarda sensu stricto* but rather represent other recently described species (*E. piscicida*, *E. anguillarum*) or unnamed clades [22,23,30]. Therefore, this section will primarily focus on *E. tarda* isolates or strains of clinical origin.

#### 4.2.1. Culture

**Figure 2:**
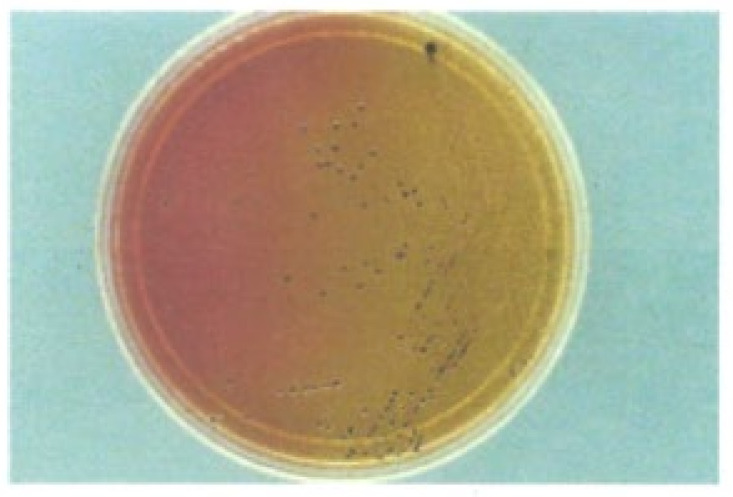
*E. tarda* colonial morphology on SS agar exhibiting black centers with clear peripheries.

There have been no systematic investigations on the plating efficiency of edwardsiellae on various selective agars, a partial reflection on the perceived low incidence of this pathogen in causing diarrhea [44]. Because of the unique biochemical features of *E. tarda* wild-type strains (H_2_S-positive, lactose- and sucrose-negative), this taxon can be easily detected on many common differential and selective enteric media when cases of bacterial gastroenteritis arise. *E. tarda* grows well on xylose–lysine–desoxycholate (XLD), desoxycholate citrate (DC), Hektoen enteric (HE), and *Salmonella–Shigella* (SS) agars producing colorless colonies on the plating medium with black centers (H_2_S-positive, Figure 2). They often resemble *Salmonella* isolates in appearance [1,104]. On MacConkey agar, *E. tarda* yields colorless transparent colonies [104]. Unfortunately, many published reports regarding *E. tarda*-associated diarrhea do not record the isolation media used in the recovery of this species from feces. When mentioned, however, the most common combinations noted are MAC/SS, MAC/HE, and MAC/XLD. Media found to be unsatisfactory for the recovery of *E. tarda* from stool include bismuth sulfite and brilliant green.

A study by Iveson [105] on diarrheal patients in Western Australia found only 22.5% of *E. tarda* strains were isolated by direct plating in contrast to the use of an enrichment broth. Enrichment broths found to be very good to excellent for the recovery of edwardsiellae include selenite cysteine, Hajna’s Gram-negative (GN), and tetrathionate broths [1,14]. Despite this, a study by Sechter [106] found tetrathionate broth unsatisfactory for the isolation of *E. tarda*. In a comparative study of enrichment broths, Iveson [105] found strontium B chloride superior for the recovery of both *E. tarda* and *Salmonella*, although this medium has not gained wide acceptance in general usage.

#### 4.2.2. Biochemical Traits

*E. tarda* is a phenotypically “tight” species, that is, most characteristics are either uniformly positive or negative for this species, similar to that observed for *Plesiomonas shigelloides*. Consistent species phenotypes in addition to those listed above for the genus include lysine and ornithine decarboxylase positivity, the presence of arginine dihydrolase, and Voges–Proskauer and urea negativity [103]. The chief biochemical characteristics useful in recovering *E. tarda* isolates from gastrointestinal contents associated with bacterial enteritis are H_2_S and indole production [102]. On selective enteric agars, picked colonies of *E. tarda* screened on Triple Sugar Iron (TSI) slants exhibit either a K/AG-H_2_S^+^ (predominant) or K/A-H_2_S^+^ reactions; lysine iron agar slants invariably produce a K/K-H_2_S^+^ reactions. Growth from TSI slants can subsequently be identified as *E. tarda* by various biochemical tests including indole positivity. On rare occasions, strains most often recovered from feces and blood can yield aberrant phenotypic properties [44]. These aberrant reactions include indole-negative isolates and strains fermenting L-arabinose, D-mannitol, and sucrose [44,103]. Table 6 lists common phenotypic characteristics of wild-type strains.

##### Biogroup 1 and Related Strains

In 1980, Grimont and associates [19] published an article describing a new species of *Edwardsiella*, *E. hoshinae*. In that study, the authors also described a group of “atypical” *E. tarda* strains with unusual phenotypic properties that included being hydrogen sulfide-negative and able to ferment several sugars including sucrose. All of these atypical strains originated from non-human sources. In 1985, Farmer and colleagues [102] named this atypical group *E. tarda* biogroup 1; all seven strains described in this publication were of animal origin. Farmer noted that no biogroup 1 strain had ever been recovered from a clinical specimen [102].

**Figure 3:**
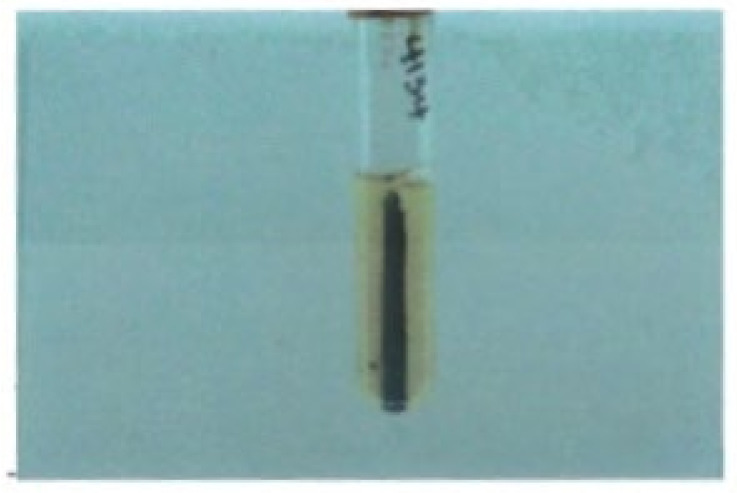
Biogroup 1-like *E. tarda* producing H_2_S on cysteine-based media.

Since then, several *E. tarda* biotype-like strains have been isolated from clinical material. Walton et al. [107] reported on a case of cholelithiasis in a 72-year-old female from whom both *E. tarda* and *E. coli* were isolated from biliary fluid. The *E. tarda* strain had some of the markers similar to biogroup 1 strains being H_2_S-negative and sucrose-positive but negative for D-mannitol and L-arabinose. Three years later, a similar strain of *E. tarda* with identical properties was recovered from the blood along with *P. shigelloides* of a patient with obstructive jaundice [108]. In both cases, the authors mentioned that while H_2_S production was not visible on TSI slants, it could be observed in one case on cysteine thiosulfate [108] (Figure 3) and in the other investigation using an API 20E strip [108]. The most recent example describes an apparent case of a liver abscess due to a biogroup 1 strain in a 26-year-old male who frequently bathed in a village pond where livestock also bathed [50]. Although reported as a biogroup 1 strain, the authors failed to provide biochemical test results.

The current data suggest that these biogroups can be isolated from clinical material and may be pathogens or copathogens in systemic infections. They may also cause gastrointestinal syndromes but may be missed because of unusual traits including negative H_2_S reactions on common laboratory media (TSI, HE). Because two of the reports displayed only some of the markers originally proposed by Grimont et al. [19], we refer to them here as “biogroup 2” isolates (Table 6). Further work in this area needs to be undertaken to identify the frequency, phenotypic diversity, and pathogenicity of these unusual strains.

#### 4.2.3. Molecular Identification

For clinical laboratories, the need to identify *E. tarda* by molecular methods is very limited. Since in most regions of the world, the apparent incidence of *E. tarda*-associated diarrhea is very low, this enteropathogen is not included on any of the FDA-approved multiplex gastrointestinal syndromic panels [109]. However, because *E. tarda* exhibits recognizable biochemical features (hydrogen sulfide and indole production), wild-type strains can easily be recognized using conventional or traditional methodologies.

There are a few instances where molecular identifications might be valuable. These situations could include confirmation of an isolate yielding a less-than-excellent identification (such as on an API 20E strip), unusual strains exhibiting atypical or aberrant reactions, or confirmation of identification by reference centers. The use of 16S rRNA gene sequencing has issues because of the high sequence similarity of many members of the *Enterobacteriales* to each other [110]. MALDI-TOF appears to be a better choice. The Bruker Biotyper identified a strain of *E. tarda* correctly to both genus and species [111]. More recent studies suggest that discriminatory protein peaks are identifiable for each of the five *Edwardsiella* species, suggesting that the use of MALDI-TOF for species identification and confirmation is appropriate [112].

### 4.3. E. ictaluri

*E. ictaluri* was initially identified as the etiological agent responsible for enteric septicemia of catfish (ESC), as documented by Hawke [20]. Following its first isolation in the United States from fingerlings of channel catfish (*Ictalurus punctatus*), *E. ictaluri* has been documented on four continents: Asia, Africa, America, and Europe, infecting as many as 44 fish species [20,21,22,23,75,86,87]. Infections caused by *E. ictaluri* can manifest itself independently of stressors and have been associated with considerable mortality rates, reaching up to 77% [15]. Nevertheless, stressors such as handling procedures, adverse environmental conditions, and high stocking densities have been observed to exacerbate mortality rates, escalating them up to 97% [42].

#### 4.3.1. Culture

Limited information is available on the isolation, agar media, and culture conditions of *E. ictaluri*. An agar medium containing colistin is effective for isolating *E. ictaluri*, which inhibits most enteric bacteria. *E. ictaluri* grows optimally at temperatures between 25 °C and 30 °C, and its growth is slow, often requiring 2–3 incubation days; colonies with a diameter of 1 mm will form [25,75]. Griffin et al. [69] reported that strains grow on Mueller–Hinton II agar supplemented with 5% defibrinated sheep blood and on static porcine brain–heart infusion broth grown for 36–48 h 28 °C. The isolation was also reported by using TSA (tryptic soy agar), EMB (eosin methylene blue), BHI (brain–heart infusion agar), and EIA (*E. ictaluri* agar) [14,28,40,44,102,103]. 

#### 4.3.2. Biochemical Traits

*E. ictaluri* is a Gram-negative pleomorphic rod that varies in length and width depending on the host [20,38,113]. It is peritrichously flagellated and has been observed to exhibit weak motility under optimal growth conditions, although strains devoid of motility have also been reported [20,114,115]. *E. ictaluri* strains are generally recognized as facultative anaerobes [116,117,118]. Biochemically, these strains demonstrate activity in catalase, ornithine decarboxylase, and hydrogen sulfide (H_2_S) production, as well as gas and acid production from glucose [15,81,119,120]. Growth on blood agar plates is slow, requiring 48 h at 30 °C to form typical colonies measuring 2 mm in diameter. *E. ictaluri* tested negative for cytochrome, urease, indole, citrate, KCN, and most sugars, such as lactose, sucrose, D-mannitol, dulcitol, and salicin. However, *E. ictaluri* tests positive for catalase, nitrate reduction to nitrite, glucose fermentation (O/F test), lysine decarboxylase, ornithine decarboxylase, and D-glucose. While some variable test results have been published, *E. ictaluri* is commonly identified by its growth conditions on agar media and main biochemical characteristics [15,28,44,65].

#### 4.3.3. Molecular Identification

16S rRNA gene sequencing was reported as a valid bacterial identification method twenty years ago, and housekeeping genes were further found to be useful for species-specific identification of closely related taxa [110]. Using the 16S gene region (named IRS) and intervening sequence (IVS) in the 23S rRNA, a method was designed to distinguish *Edwardsiella* genus from other genera and *E. ictaluri* from *E. tarda*, respectively [121]. Some authors have demonstrated that the IRS gene region has no positivity except for the *Edwardsiella* genus, and IVS was a more specific and valuable gene region with the presented primers to identify *E. ictaluri* from *E. tarda* and tested genera [121]. 

One of the main housekeeping gene regions named *gyr*B and *sod*B have been reported to identify *E. ictaluri* with a high discrimination value, such as 99.73% and 99.81%, respectively [112]. Different from mostly used housekeeping genes, the upstream region of the fimbrial gene named EDi, with a predicted product size of 470 bp, has been documented with a valuable identification between *E. ictaluri* and typical *E. tarda* and atypical *E. tarda* strains [122]. 

### 4.4. E. piscicida

Following its initial differentiation from *E. tarda*, there has been a notable surge in the documentation of *E. piscicida*. Genetic investigations conducted on *E. tarda* isolates from historical records have indicated that numerous isolates formerly categorized as *E. tarda* are now classified as *E. piscicida* [23,30,112]. This recent reclassification and retrospective analysis of archived data imply that *E. piscicida* poses a more substantial challenge in global finfish aquaculture compared to *E. tarda*.

#### 4.4.1. Culture

*E. piscicida* shows capacity to grow across a wide range of general growth media, such as Trypticase^TM^ soy agar, brain–heart infusion agar, Mueller–Hinton agar, Luria broth (LB), marine agar, tryptic soy agar supplemented with 5% sheep blood, or blood agar containing 5% bovine blood [21,28,40,44,89,102,103]. The bacteria can grow in aerobic and anaerobic conditions between 25 °C and 37 °C but not at 12 °C and 42 °C unlike the *E. tarda* type strain (ATCC 15947^T^) and fish isolate NCIMB 2034 for a 24 h incubation period. 

#### 4.4.2. Biochemical Traits

Colonies obtained from both the *E. tarda* type strain (ATCC 15947^T^) and a singular fish isolate (NCIMB 2034) exhibited circular, convex morphology, accompanied by a distinct narrow β-hemolytic zone, minimally extending beyond the colony periphery after 24 h of incubation. Under anaerobic conditions, all strains displayed growth, yielding pinpoint colonies within the same timeframe. In LB broth supplemented with 3% and 5% NaCl, both *E. piscicida* fish isolates and the type strain demonstrated growth, while no growth was observed in solutions containing 6% NaCl or higher concentrations. *E. piscicida* demonstrated growth within a temperature range of 16 °C to 37 °C, with no growth observed at temperatures of 12 °C or 42 °C. All strains exhibited motility and tested negative for cytochrome oxidase, citrate, D-mannitol, L-arabinose, lactose, L-rhamnose, D-sorbitol, and trehalose. Conversely, they tested positive for catalase, indole production, H_2_S production, methyl red, lysine and ornithine decarboxylases, and gas production from D-glucose (Table 7) [21,28,88,102,103,123,124,125].

#### 4.4.3. Molecular Identification

Recent advances in molecular techniques have enabled the delineation of closely related species of pathogenic bacteria, such as *E. tarda* and *E. piscicida* [21,23,126]. Notably, *E. anguillarum* and *E. piscicida* are absent from biochemical databases associated with the systems utilized in this study [112]. The increasing adoption of genomic technology and the consequent identification of new bacterial taxa present challenges in managing phenotypic databases, resulting in a lag in prokaryotic databases compared to evolving systematics [27]. The suitability of 16S rRNA for bacterial identification has long been debated, primarily due to the substantial sequence similarity between closely related species, the absence of unequivocal intraspecific dissimilarity values, and the lack of universal guidelines [110,126]. In such instances, alternative reference genes should be considered. The single-copy *gyr*B gene, encoding the ATPase domain of DNA gyrase, is indispensable for DNA replication and is universally present in prokaryotes [112]. It harbors conserved motifs that facilitate the design of genus- or family-specific primers [127]. The *gyr*B gene has been proven instrumental in elucidating the diversity of a broad spectrum of bacteria and exhibits greater resolution than 16S rRNA in discriminating closely related members of the *Enterobacteriaceae*, including *Edwardsiella* spp. [23,30,125].

The efficacy of *gyr*B in the classification and identification of *Edwardsiella* has been corroborated elsewhere [128,129,130], and the findings presented here further endorse the utility of *gyr*B as a suitable marker for distinguishing *Edwardsiella* species. DNA *gyr*B and *sod*B were the most reported gene regions for the identification of *E. piscicida* from the closest species, such as *E. ictaluri* and *E. tarda*, which had a discriminatory power of 99.78% and 99.97%, respectively [112]. In addition to most of the successful PCR methods developed for the identification and discrimination of *E. piscicida*, Sakai et al. [122] also designed valuable and discriminatory qPCR primer probes named EP14529F, EP14659R, and EP14615P, which encode the gyrB gene region previously proposed by Griffin et al. [129].

## 5. *Edwardsiella* Infections in Humans and Animals

### 5.1. Human Infections

As with many Gram-negative enteric pathogens, the range of human illnesses and syndromes associated with *E. tarda* infections is substantial. Unfortunately, due to the perceived low frequency of edwardsiellosis in most countries, no retrospective or prospective studies encompassing all *E. tarda* infections from a single medical institution over protracted periods of time have ever been published. This includes regions of the globe including SE Asia where the expected incidence of infection should be higher due to culinary, dietary, and occupational habits and professions. Bockemühl et al. [11] reported on 25 case histories of edwardsiellosis from six different laboratories in Thailand in 1971 (no time frame). Twenty-three (92%) of these specimens were from stool or rectal samples and one each (4%) from blood and bile. Reviews on selective *Edwardsiella*-associated syndromes, such as bacteremia, have been reported, but comprehensive investigations are still lacking.

**Figure 4:**
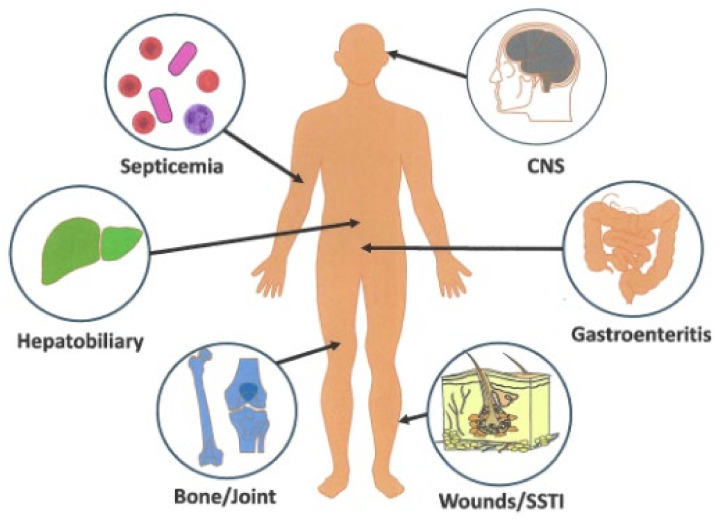
Schematic representation of the major intestinal and extraintestinal syndromes associated with *E. tarda* infections; SSTI, skin and soft tissue infections.

*E. tarda* infections can be broken down into two broad groups, namely intestinal and extraintestinal infections [14,44] (Figure 4). Intestinal infections invariably involve episodes of gastroenteritis or related sequelae; >80% of all scientific or medical *E. tarda* publications involve this syndromic disease [14,44]. Two retrospective investigations of *E. tarda* extraintestinal infections from Taiwan (~3 years) and a university-based teaching hospital on the Gulf Coast (10 years) found the predominant sites of *E. tarda* infection to be the biliary tract liver abscess (41%) in one and soft tissue infections (STI, 60%) in the other, respectively [131,132].

#### 5.1.1. Gastrointestinal Syndromes

Issues in understanding the infectious complications, disease-associated epidemiology, and laboratory complexities involving the diagnosis of *E. tarda* gastroenteritis mirror or parallel findings highlighted above for all human cases of edwardsiellosis. In particular, our knowledge of *E. tarda* gastroenteritis suffers from (1) the observed low incidence/prevalence of the disease in most countries, (2) lack of epidemiologic surveys assessing the frequency and type of *E. tarda* diarrhea from presumed endemic areas (subtropical), (3) few reported case histories of diarrhea since 2000, (4) commercially available syndromic gastrointestinal panels without a *E. tarda*-specific molecular target, and (5) medical reviews of acute or emerging causes of bacterial gastroenteritis that do not list infective agents other than major enteropathogens.

Although most diagnostic microbiology laboratories recognize *E. tarda* as a human enteropathogen, there is still some skepticism in the medical community as to whether this bacterium is a legitimate enteric pathogen. Data supporting this viewpoint stem from early studies conducted in the 1960s and 1970s indicating *E. tarda* can be isolated from asymptomatic individuals as well as from diarrhetic stools often in conjunction with recognized enteropathogens, such as *Shigella flexneri* and *Entamoeba histolytica* (Table 8). Furthermore, a defining characteristic of most gastrointestinal pathogens is the ability to cause diarrheal outbreaks, which is lacking in the case of *E. tarda*, although a cluster of eight asymptomatic persons at a day-care center in Florida in 1990 has been reported [133].

Despite these limitations, the vast preponderance of evidence indicates *E. tarda* is a bona fide gastrointestinal pathogen [44]. This evidence includes the facts that (1) *E. tarda* is rarely recovered from the gastrointestinal contents of healthy persons with ratios of infected/colonized individuals exceeding 3:1 [14] (Table 8), (2) well-documented case reports of severe gastrointestinal illnesses including chronic enterocolitis with *E. tarda* as the only potential pathogen, (3) patient immune responses (agglutinating antibodies) to infecting strains weeks to months post-infection [14,50,134], (4) episodes of *Edwardsiella* septicemia immediately following diarrheal episodes where *E. tarda* was isolated from stool, and (5) resolution of diarrhea subsequent to treatment and eradication of *E. tarda* from feces [55,135].

*E. tarda* is not a normal inhabitant of the human gastrointestinal tract, although occasional investigations have found higher rates of *E. tarda* carriage in controls versus symptomatic persons located in rural communities in Panama [93]. Multiple early pioneering studies assessing the fecal carriage rate of *E. tarda* in healthy persons or control groups estimated the frequency to range between 0% and 0.8% (Table 8). One Japanese study by Onogawa and others in 1976 [136] surveying over 255,000 healthy children and 97,000 food handlers found the asymptomatic colonization rates to be 0.01% and 0.001%, respectively.

**Table 8:** Early studies on the association of *E. tarda* with gastroenteritis.

Study	Country	Study Period	Population	Disease Presentation	*E. tarda* Prevalence (%)		Co-Pathogens Present ^a^
					Patients	Controls	
Bhat et al. [137]	India	1963–1965	Rural, Urban	Juvenile diarrhea	0.48	0	25 (%)
Gilman et al. [138]	Malaysia	NG	Orang Asli	Bloody diarrhea	13.9	0.8	86 (%)
Iveson et al. [106]	W. Australia	NG	Aboriginal	AGE	0.3	NG	27.5%
Makulu et al. [139]	Zaire ^b^	1965–1972	Zaïrese (88%) Europeans (12%)	AGE	0.25	0	49%
Kourany et al. [93]	Panama	1965–1972	Urban, Rural	AGE	0.33	0/0.66 ^c^	10%

Abbreviation: AGE, acute gastroenteritis; NG, not given. ^a^ percentage of recognized copathogens present with *E. tarda*. ^b^ Zaïrese patients (88%); European patients (12%). ^c^ urban/rural.

Gastroenteritis associated with intestinal *E. tarda* infection can run the gamut of symptomatology linked to this disease syndrome. Originally, three main *E. tarda* disease states were described, namely (i) gastroenteritis, (ii) a typhoid-like illness, and (iii) an asymptomatic colonization condition [44]. Currently, the most common clinical presentation of *E. tarda* diarrhea is acute secretory gastroenteritis or enteritis. Acute gastroenteritis typically manifests itself as a mild watery diarrhea of 3 to 5 days duration. Stools are often noted to be yellow/green tinged, sometimes with mucus [52,140]. A low-grade or intermittent fever (37.8 to 38.3 °C) may occasionally accompany such presentations as well as abdominal pain, cramps, and nausea [44]. Bowel movements can range from 4 to 5/day but evacuations as high as 10 to 20/day have been recorded [44,55,141]. This form of the disease has commonly been described in young infants or children but also occurs in adults and in long-term native inhabitants of such remote locales as the Orang Ali of West Malaysia [138] and aboriginal children in northwest Australia [105]. 

From often-self-limiting enteritis, specific episodes of *E. tarda* can progress to a more severe illness, including enterocolitis, bloody dysentery, or even mimicking typhoid fever on occasion. Dysentery or bloody diarrhea has been described on a few occasions as part of an epidemiologic survey or as individual case reports. In a series of gastrointestinal infections, Bockemühl et al. [11] reported two cases of *E. tarda*-associated dysentery. One case involved a 37-year-old woman who was also positive for *S. flexneri*; however, a second case of dysentery was observed in a 20-year-old female that yielded *E. tarda* as a sole pathogen. Marsh and Gorbach [135] described a case of *E. tarda* enterocolitis in a 31-year-old person who presented with bloody diarrhea. A case of typhoid fever-like illness in a 46-year-old man who had returned from a trip to Mexico was reported in 1980 by Clarridge and coauthors [142]. He developed a variety of symptoms including lower abdominal pain, nausea, minimal diarrhea, and fever. The presumptive diagnosis was typhoid fever, and *E. tarda* was isolated from both his blood and feces.

In addition to the traditional symptoms listed above, several other gastrointestinal conditions have been linked to *E. tarda* infection. Chronic gastroenteritis is usually defined as diarrhea persisting for more than 14 days. Several cases of chronic gastroenteritis have been reported to be caused by edwardsiellae. A 12-year-old boy who had consumed raw shrimp and fish while traveling in Japan developed watery diarrhea with mucus that lasted for three weeks before seeking medical attention [52]. His stool culture yielded *E. tarda* O4:H4. Similarly, *E. tarda* was isolated from a stool culture of a 72-year-old woman with severe diarrhea, cramping abdominal pain, and low-grade fever for a three-week duration after consuming raw oysters [143]. A more pronounced case of chronic enteritis was reported by Chida and others [141] in a 12-year-old Japanese boy who had consumed grilled eel. His symptoms had persisted for 3 months before his hospital admission. Over that time span, he had presented with a variety of symptoms, such as watery diarrhea (10X/day), abdominal pain, fever, a bloody stool (once), nighttime diarrhea, and weight loss. A stool culture analyzed upon admission yielded a final diagnosis of *E. tarda* gastroenteritis. Finally, a protracted case of mild-to-severe intermittent diarrhea of >5 months duration was seen in a 53-year-old male who had spent the past 30+ years continuously in Central and South America. Fecal examination demonstrated rare *Trichurus trichuria* ova and culture grew *E. tarda* [134]. An immune response (1:160 agglutination) to the infecting strains 5 months after his bout of severe diarrhea suggests a causative role for this agent in his gastrointestinal disorder.

In addition to causing gastroenteritis, *E. tarda* has been associated with triggering or fomenting several idiopathic inflammatory bowel conditions. These conditions include Crohn’s disease (CD) [144,145] and ulcerative colitis (UC) [146,147]. In an eight-year period, Koido and colleagues [146] identified nine cases of UC relapsing from a quiescent state to a mild-to-severe form due to *E. tarda*. For both CD and UC, the vehicle of infection appeared to be the consumption of contaminated or raw freshwater fish [144,145,146].

#### 5.1.2. Septicemia

The most serious life-threatening extraintestinal complication of *E. tarda* infection is septicemia, with or without secondary manifestations or sequelae [46,47]. While one recent case report purportedly documents *E. ictaluri* bacteremia in a Nigerian child, the authors fail to provide laboratory data to support this identification other than routine, conventional tests such as API 20E, which are inadequate [148]. To date, all bona fide cases of *Edwardsiella* bacteremia have been caused by *E. tarda*, analogous to the situation listed above for intestinal infections.

Our knowledge of *E. tarda* sepsis has changed significantly over the past decade. Earlier reports on a limited number of cases (16–28) of *E. tarda* bacteremia listed a mean age of infection as 40.4 years with reported mortality rates of 32 to 47% [14,44]. Recent Japanese investigations have greatly expanded our reference point of knowledge concerning bloodborne edwardsiellosis [46,47,149]. Hirai and coinvestigators [46] reviewed 77 cases of *E. tarda* bacteremia in the medical literature between 1968 and 2013. They found the median age of infection to be 61 years (range, 2 mo.–101 years.) with an M/F ratio of 1.6; the observed mortality rate in this study was 44.6% [46]. An 11-year retrospective epidemiologic investigation (2005–2016) of *E. tarda* septicemia at a 1166-bed tertiary-care hospital in Japan found the median age of 26 patients was 75 years with an M/F ratio of 1.0 [149]. The overall 30d and 90d mortality rates were 12% and 27%, respectively. Finally, another single-institution retrospective Japanese study (2005–2022) found the median age of persons septic with *E. tarda* to be 77.5 years with an M/F ratio of 1.3 [47]. Gross mortality rates at 30d and 60d were 9% and 26%, but most deaths were due to underlying conditions such as malignancy; attributable death rates due to *E. tarda* varied from ~3–7% in this report [47]. One study found a higher *E. tarda* mortality rate (61%) associated with soft tissue infections [46]. The collective results of these studies suggest the average age of clinical cases of *E. tarda* sepsis is increasing (>65 years) while the observed mortality rate is declining. 

Clinical signs of *E. tarda* sepsis are similar to those of other Gram-negative septicemias. Prominent features commonly include fever, concurrent or antecedent diarrhea, abdominal or epigastric pain, hypotension, and chills [44]. The fulminant disease can include septic shock with edema, bullae, and ischemia on the extremities [48,150,151,152]. 

Persons prone to developing *Edwardsiella* sepsis (80–90%) include individuals with underlying comorbid or immunocompromised conditions. Major underlying conditions associated with sepsis include solid tumors or hematologic malignancies (59–66%), gallstones (45%), and cirrhosis of the liver (17%) [46,47]. Other less common disorders linked to edwardsiellae bacteremia include diabetes mellitus [14,44,153]. Two unusual syndromes have been reported on multiple occasions predisposing people to *E. tarda* septicemia. Cushing’s syndrome or a cushingoid-like condition has been described in two cases of *E. tarda* bacteremia occurring in an 18-year-old man and a 20-year-old woman [154,155]. In both instances, multiple organ sites were involved including the liver and peritoneum in addition to blood. A second condition associated with *E. tarda* sepsis comprises disorders sometimes linked to iron overload states such as sickle cell anemia or hemoglobinopathy, neonatality, leukemia, and cirrhosis [13,14,44,46,156]. Approximately 15% to 17% of episodes of *E. tarda* arise in healthy persons with no known underlying disease [46,149].

In addition to medical complications, several risk factors have been associated with *E. tarda* septicemia. These factors include alcoholism, consumption of raw consumable products, and exposure to marine water or animal feces. A 2015 review identified one of these three risk factors in 29.5% of cases of edwardsiellae bacteremia [46]; other studies have reported similar values [47]. Many patients presenting with *E. tarda* septicemia present with multiple comorbid conditions or risk factors. Healey and coauthors [48] described a fatal case of *E. tarda* septicemia in a 59-year-old woman with lung cancer, cirrhosis of the liver, and alcohol abuse. One day prior to her being transported to the Emergency Department of a Florida hospital, she consumed raw oysters and subsequently complained of generalized abdominal pain the next morning. Similarly, LeBlond [150] described a fatal case of *E. tarda* bacteremia in a 58-year-old man related to a catfish bite. He had multiple underlying medical conditions including cancer, diabetes, and alcohol abuse.

Two studies have estimated the frequency of *E. tarda* bacteremia in positive blood cultures to vary between 0.1% and 0.4% [47,149]. The majority (~90%) of these blood culture-positive specimens involve monomicrobic bacteremia [14]. Polymicrobic infections, however, are not uncommon. One survey found 27% of *E. tarda* bloodstream infections to be polymicrobic in nature [149]. Positive *E. tarda* blood cultures should alert physicians to the possibility of from 1 to 4 additional coinfecting agents depending upon the clinical diagnosis (liver abscess or secondary peritonitis). Coinfecting agents fall into two main groups. The first group involves microbes typically residing or associated with the human microbiome including Gram-negative enteric bacilli, streptococci, enterococci, and anaerobes [47,149]. Due to the fact *E. tarda* is considered an aquatic species [157] organisms inhabiting both marine and freshwater environs must also be considered as possible copathogens. Wang et al. published [131] a case of polymicrobic *E. tarda* bacteremia in a 61-year-old woman who had consumed shark meat the day before hospitalization. *E. coli* and *Shewanella putrefaciens* were recovered from her blood in addition to *E. tarda*. In approximately 40% of septicemias, *E. tarda* is also found at other anatomic sites, particularly the liver [14,50,60,151]. 

#### 5.1.3. Wound Infections

Currently, the third most common *E.* tarda-related illness reported in the medical literature is wound infections. *Edwardsiella*-associated wounds can span the spectrum of soft tissue complications ranging from mild cases of cellulitis [131,158] to episodic myonecrosis [45] or devastating necrotizing fasciitis [62]. In many instances, the clinical significance of such isolations is often unknown or can be difficult to interpret from both a medical and laboratory standpoint due to the concurrent recovery of other Gram-positive or Gram-negative bacteria from infected tissue [159] and the limited availability of medical information.

There are two types of *Edwardsiella* wound infections, each differing from one another in a few ways including source of infection, routes of transmission, and patient medical histories. These two types of wound infections include (1) soft tissue infections (STIs) resulting from trauma and (2) pus-filled cavitary lesions [44].

*E. tarda* STIs most often present with pain, swelling, erythema, and/or a purulent discharge from an extremity including the hands, feet, legs, and arms [131,132]. The precipitating event leading to such infections is either a laceration resulting from an accident (fall) or a penetrating injury with exposure to edwardsiellae residing in aquatics environs. The most common vehicles reported to cause such infections are catfish spines or fish bones [45,132,160,161]. Other aquatic-associated injuries leading to infection involve occupations (fisherman) or recreational activities (swimming, diving, crabbing, shellfish) [45,132,134,162]. However, rare *E. tarda* infections have occurred from injuries sustained from glass or car doors with no apparent association with water [45]. Most infections presenting in this manner occur in healthy males < 30 years of age [45,160,162]. Infections can progress from the initial trauma site depending upon the delay in time seeking medical attention, severity of the wound, host immune status, and infectious dose of the pathogenic strain. Wound cultures often contain other aquatic bacteria (particularly *Aeromonas hydrophila* and *S. putrefaciens*) complicating the etiologic diagnosis of the disease, but further attesting to the reservoir of infection [45,162]. Initial treatment may require incision, drainage, and antimicrobial therapy; however, more severe STIs (necrotic fasciitis) can necessitate debridement and in rare cases amputation [62,161]. For older patients with underlying medical complications, which include alcohol abuse, cirrhosis, and diabetes, the pathway leading to wound infection may not involve a traditional penetrating trauma but rather the consumption of raw fish or seafood [62,158]. In this instance, multiplication in the gastrointestinal tract prior to translocation into the circulatory system leads to extraintestinal disease [62].

In contrast to STIs, *E. tarda* cavitary lesions leading to abscess formation appear to predominate in women. Common presenting symptomatology includes fever, severe abdominal or right-quadrant pain, and nausea/vomiting. Most abscesses occur in older persons with one or more underlying comorbid conditions including hepatobiliary disease, cancer, and diabetes [60,132,151,163]. Unlike many STIs stemming from penetrating injuries, abscess formation appears to result from an endogenous septic process originating from the ingestion of raw/fresh fish or shrimp [60,163]. In these scenarios, blood and stool cultures, in addition to abscess samples, are often positive for *E. tarda*. Abscess cultures often yield a pure culture of *E. tarda* but polymicrobic infections have been reported [159].

*E. tarda* cavitary lesions have been described at multiple anatomic sites but the most common of these are tubo-ovarian and liver abscesses [44,45,50,60]. Ota [60] reviewed eight cases of *E. tarda* liver abscess formation. Of these eight individuals, half had no underlying illnesses while three had hepatobiliary disease. Such infections when presenting to an emergency room or hospital can progress rapidly to disseminated intravascular coagulation syndrome [163] or fulminant septic shock [151]. The reported fatality rate is 38%.

#### 5.1.4. CNS Illnesses

Although extremely rare, cases of central nervous system (CNS) disease attributed to *E. tarda* date back to almost the inception of the genus with the description in 1968 of a fatal case of septicemia and meningitis in a 31-year-old splenectomized woman with SLE [8]. Since that report, only a handful of CNS cases have been published over the intervening 50+ years. These CNS cases include subdural hematoma [164], neonatal brain abscesses [165], meningoencephalitis [166], and the most common *E. tarda* neurologic complication, meningitis. 

There have been seven published cases of *E. tarda* meningitis including one case of meningoencephalitis [13,166,167,168]. Four cases occurred in adults and three in neonates. Adult cases (17 to 78 years) have presented in persons with fever, headache, and altered mental status who are either compromised because of steroid therapy systemic lupus erythematosus [8] or have other predisposing conditions to infection such as cirrhosis due to excessive ethanol abuse or eating raw fish [166]. In contrast, neonates suffer from prematurity (33–35 weeks), low birth weight, and failure to thrive [8,168]. A definite source of infection in these infants is not identifiable. In all these infections, both blood and CSF are culture-positive for *E. tarda*. The overall mortality rate for these seven cases is 57% (adults 50%, neonates 67%).

#### 5.1.5. Miscellaneous Infections

In addition to hepatobiliary disease being associated with *E. tarda* bacteremia [50,60,62,107,169], a variety of other rare complications have been attributed to edwardsiellosis. These maladies include peritonitis [51], urinary tract infections [44,56], respiratory tract disease [59], and endocarditis [170]. While scarce, bone and joint infections caused by *E. tarda* have been described on several occasions and associated with arthritis of the knee, thoracic spondylitis [58], and osteomyelitis [171]. Finally, a 2023 communication by Higashigawa et al. [172] documents a series of published and unpublished (abstracts) reports on *E. tarda* as an important cause of maternal–fetal infections in Japan. These infections may again be related to food preference and pose an important health risk to pregnant women.

### 5.2. Piscine and Animal Infections

#### 5.2.1. Fish Diseases

The global aquaculture production of various fish species, such as tilapia, salmon, and carp, has continuously increased in the last decade, but bacterial infections are causing high mortality. Aquaculture facilities constructed in water sources at temperatures above 20 °C have suffered significant economic losses due to outbreaks of edwardsiellosis [67]. Environmental factors such as high temperatures, poor water quality, and organic debris trigger *Edwardsiella* infections in fish. These infections are most common from spring to early autumn when water temperatures range from 22 to 28 °C, particularly in tropical and subtropical regions [74,78,79,80,81,82,83,84,85].

As mentioned in previous sections, *Edwardsiella* species have been reported in many fish species, especially ictalurid, piscine, and *Anguilla*. *E. piscicida* has been reported as far back as 1979, and the agent is commonly reported with congestion of the fins, hemorrhages and erythema in the ventral skin, exophthalmia, discoloration of the skin, external hemorrhages and petechia in musculature, erratic swimming, bottom-dwelling, pallor and mottling of the liver, splenomegaly, granulomas in internal organs, ascites, deep abscesses, gastrointestinal septicemia, granulomas in liver, ascites, necrosis in liver and kidney, white patches of mucus, and opaque corneas [76,88,125,129]. In *E. tarda* infections, watery and bloody ascites in the abdominal space and reddish foci on the skin, enteritis, liver congestion and vent protrusion, enlargement in the spleen and kidney, abscess-like lesions filled with a purulent fluid in the kidney, lethargic and frequently swam off balance, exophthalmia, the opacity of the eyes are commonly reported as symptoms and/or lesions [173,174,175]. In some cases of *E. tarda*, disease manifestations mimic the symptoms of *E. ictaluri* [128]. 

*E. ictaluri*, also known as ‘enteric septicemia of catfish’ (ESC), is a pathogen that affects over 44 fish species, although it primarily infects fingerlings of channel catfish (*Ictalurus punctatus*) in the United States aquaculture industry [15]. The classic lesion caused by *E. ictaluri* is a ‘hole in the head’, which is a result of the digestion of the cartilaginous skull cap [176]. The septicemic form of the disease is easily recognizable by the presence of hemorrhagic lesions on the jaw and occasionally around the base of the fins [84,114,119,123]. Bloody serous fluid is often present in the body cavity. Enlarged spleens, as well as petechial hemorrhages in the liver, visceral fat, and intestine, are common symptoms of this form of the disease [176,177,178].

*E. anguillarum* comprises a cohort of genetically distinct isolates originating from eels and other brackish water fishes, which diverge significantly from other *Edwardsiella* lineages. Despite its genetic dissimilarity from other *Edwardsiella* species, infections attributed to *E. anguillarum* predominantly manifest symptoms such as redhead, congestion, and increased size and redness of the pectoral, gluteal, and abdominal fin [31,179]. Affected individuals exhibit enlarged, darkened, and bluish livers, with severe cases presenting ulcerative lesions in the liver and kidney, as well as later-stage liver ulcers, mucus-filled intestines, and swollen anus lesions/symptoms [89].

*E. anguillimortifera* and *E. hoshinae* infections in fish have not been extensively documented, including their symptoms and lesions. *E. anguillimortifera* was previously classified as a species of *E. tarda* and may share similar pathogenic characteristics [29]. The habitat of *E. hoshinae* remains poorly understood. *E. hoshinae* has been isolated from various animals, including monitor lizards, puffins, an unspecified lizard, a flamingo, and water [19,44,65,129,130]. However, its effects on fish have not been extensively studied. All *Edwardsiella* species show a septicemic form (acute phase) and ulcerative form (chronic phase) of infection. In the acute form, the symptoms/signs mentioned for each infection are seen, while in the chronic form, chronic progressed abscesses, nodules in the skin and internal organs, and necrosis in tissue, muscle, and organs were reported [46,67].

#### 5.2.2. Outbreaks

Infection with *Edwardsiella* species often leads to the development of systemic disease, and outbreaks have caused enormous economic losses in more than 20 commercially important fish species worldwide since 1962 [130]. For both *E. ictalurid-* and *E. tarda*-associated infections, morbidity and mortality are known to increase with increasing water temperature. Other factors that may increase the incidence of infection include stress and pollution. However, in some reports, it is documented, along with species such as *Aeromonas*, *Flavobacterium*, and *Pseudomonas*, that the number of outbreaks due to *Edwardsiella* has been increasing, especially in regions where water temperatures are rising in ponds where fish are present [104,162,180]. Reports of *Edwardsiella* outbreaks usually include *E. tarda* and *E. piscicida* species. However, high mortality rates due to *E. ictaluri* and *E. anguillarum* species have also been reported in recent years. While there are many reports of mortality due to *Edwardsiella* species, there is insufficient information on the mortality rates caused by these agents alone. In natural outbreaks caused by *E. ictaluri*, mortality rates have been reported to be as high as 50%, while reports of *E. tarda* mortality have been as high as 80% (Table 9). 

#### 5.2.3. Vertebrates and Other Groups

*Edwardsiella*-associated outbreaks in fish have been on the rise in recent years. However, there is a lack of research on severe mortality reports and the associated pathogenesis in vertebrates other than fish, despite reptiles being identified as natural reservoirs of *Edwardsiella* species [69,185,186]. It is noteworthy that *E. tarda* infections have been documented in alligators, crocodiles, lizards, tortoises, aquatic turtles, and snakes. *E. tarda* has been shown to cause systemic infections and bacteremia in immature and hatchling Siamese crocodiles, resulting in a range of negative health effects such as pilling up, asphyxia, mouth sores, skin diseases, gastropathy, and fungal and bacterial infections [187]. Furthermore, histopathological effects, including necrotic hepatitis, hemorrhagic nephritis, fibrinous pneumonia, and necrotic splenitis, have been observed. These findings demonstrate the significant impact that *E. tarda* can have on the health of Siamese crocodiles. *Edwardsiella* may not have extensive documentation on mortality rates as reported in fish outbreaks, but it has been shown to cause individual mortality and typical lesions in other animals. For instance, *E. tarda* infection in a female grass snake resulted in low body condition and multiple subcutaneous nodules commonly associated with edwardsiellosis [99]. *E. tarda* generated mortality rates of over 60% in Chinese soft-shelled turtles and significant mortalities in various other turtle species [188]. Additionally, a 21-year-old male Eurasian brown bear (*Ursus arctos arctos*) experienced sudden death, characterized by a swollen abdomen with hemorrhagic congestions of the gastroenteric organs, ascites, and hemorrhagic exudates around the mouth [189]. These findings indicated a sudden and severe illness that led to the bear’s unfortunate demise. The cause of death for the ducks and egrets was gastrointestinal lesions and septicemia resulting from *E. tarda* infection [101]. Isolation from various Antarctic wildlife, including southern giant petrels, brown skuas, south polar skuas, kelp gulls, greater sheathbills, chinstrap penguins, and eggs of Adelie penguins and Weddell seals, accounted for 15.1% of the 1855 samples collected of the Antarctic wildlife samples [100].

## 6. Pathogenicity

*Edwardsiella* species can survive in various external environments, such as pond water and mud, thriving under rising water temperatures and increased organic matter due to higher feeding rates and larger amphibian populations. This bacterium can exist in a carrier state in diverse hosts, including mammals, birds, reptiles, amphibians, and aquatic invertebrates. Under adverse conditions, these bacteria may become non-culturable yet remain viable, undergoing morphological changes from short rods to coccoid forms. When such cells are revived under experimental conditions, like within chick embryos, they can regain their typical morphology and infectious capabilities, potentially leading to infections and mortality in hosts such as trout [67].

The pathogenicity of *Edwardsiella* is multifaceted, driven by complex factors, including poorly understood mechanisms of how the bacteria attach to and penetrate host tissues, typically through the intestines and abraded skin areas. Studies have noted the bacterium’s ability to adhere to and penetrate epithelial cells, underscoring its virulent nature [190]. Moreover, *Edwardsiella* has a common molecular switch that allows it to adapt to free-living and host-associated lifestyles. This includes its survival as planktonic cells within biofilms or as intracellular and extracellular organisms within hosts [69,77]. Leung et al. [33] highlighted a crucial question: how do these typically non-virulent, free-living isolates lacking significant virulence genes like EsrB and the T3SS/T6SS clusters transform into pathogenic forms capable of a host-associated lifestyle? This transition is critical to understanding the dynamics of infection and disease propagation within aquatic and terrestrial environments.

### 6.1. Virulence Factors

Pathogenic *Edwardsiella* species are particularly notable for carrying critical virulence genes, including the Type III, Type IV, and Type VI secretion systems (T3SS, T4SS, and T6SS) [43]. These systems are instrumental in the bacteria’s ability to infect and cause disease in hosts. The T3SS allows bacteria to inject effector proteins directly into the host cells, manipulating the host’s cellular processes to the advantage of the pathogen. This system is often likened to a molecular syringe through which the bacteria can directly influence host cell activities, facilitating infection and immune evasion. The T4SS is similarly critical but is more versatile and involved in the translocation of virulence factors and the transfer of genetic material between bacterial cells, which can include the transfer of antibiotic resistance genes. This system increases the adaptability and resilience of *Edwardsiella* in varied environmental conditions. The T6SS, like a molecular spear, is used by the bacterium to deliver toxic molecules directly into neighboring cells, which can be either eukaryotic host cells or competing bacteria. This system plays a key role in bacterial competition and survival within the host, contributing to the pathogen’s virulence and ability to colonize host tissues effectively [16,33,41,43,67,77,190].

These secretion systems underscore the sophisticated mechanisms at play in *Edwardsiella* pathogenesis, enabling these bacteria to invade hosts, evade immune responses, and establish infections, which can lead to significant disease outbreaks in aquaculture settings. Some of the most reported virulence genes and their mechanisms were presented in Table 10 for different *Edwardsiella* species. 

### 6.2. Virulence Factors That Have Been Associated with Human Disease

Edwardsiellosis is influenced by a complex interplay of environmental conditions and specific bacterial virulence factors. Key factors proven to contribute to the disease include (1) Environmental Stress: *Edwardsiella* species thrive in varying environmental conditions, such as changes in salinity, temperature, and nutrient availability. These conditions can induce stress responses in bacteria, enhancing their pathogenic potential; (2) Biofilm Formation: The ability to form biofilms is a critical virulence trait of *Edwardsiella*, allowing it to adhere to surfaces and protect itself from hostile environmental conditions and the host’s immune responses; (3) Host Immune Evasion: The pathogen has developed mechanisms to evade and suppress the host immune system, facilitating persistent infections and systemic spread within the host; (4) Horizontal Gene Transfer: The acquisition of virulence and antibiotic resistance genes through horizontal gene transfer significantly contributes to the pathogenicity and resilience of *Edwardsiella* in both environmental and host-associated settings [16,33].

### 6.3. Animal Studies or Models of Infection

Experimental studies on the pathogenicity of *Edwardsiella* species in fish, specifically targeting *E. tarda*, *E. piscicida*, and *E. anguillarum*, reveal significant insights into their disease-inducing capabilities. *E. piscicida* demonstrates notably higher virulence in catfish, causing severe systemic infections that can lead to substantial mortality. In contrast, *E. tarda* and *E. anguillarum* show reduced pathogenicity in similar settings [31]. These findings highlight the critical nature of *E. piscicida* as a major pathogen in aquaculture, necessitating focused measures for management and control. Under experimental conditions, the differential pathogenicity among these species underscores the importance of accurate identification and understanding of their specific interactions with host species [31]. The suitable experimental fish challenge model for *E. ictaluri* infection is Nile tilapia and for *E. tarda* Channel Catfish, which has been reported to illuminate disease pathogenesis and immune response in Edwardsiellosis cases [38,191]. 

In the experimental studies using zebrafish as a model organism, immersion infection with *E. tarda* provides a straightforward technique for inducing disease and inflammatory responses in zebrafish embryos. This method has proven effective in elucidating the pathogenic mechanisms of *E. tarda*. Furthermore, studies extend to adult zebrafish, which are susceptible to *E. tarda* infections. Upon exposure, adult fish exhibit significant production of inflammatory cytokines, underscoring the robust immune response triggered by this pathogen. The findings highlight the utility of zebrafish as a versatile model for studying bacterial infections and host–pathogen interactions in aquatic organisms [192].

Recent research noted the utility of goldfish as an experimental model to explore the pathogenesis of *E. piscicida*. The findings reveal that goldfish are susceptible to *E. piscicida* infection, displaying dose-dependent mortality and significant bacterial replication within vital organs early post-infection. This model has proven effective for studying not only *E. piscicida* infections but could also be extended to other aquatic pathogens, enhancing our understanding of fish diseases [193].

A different animal model for studying bacterial enteritis in farmed seahorses using *E. tarda* isolated from the *Hippocampus erectus* has been developed. This model utilizes two bacterial concentrations to induce enteritis via intraperitoneal injection, effectively demonstrating the disease’s impact on growth inhibition and significant weight loss alongside typical symptoms like anorexia and anal inflammation. The model also employed a robust evaluation system using 19 indicators across external, histological, and molecular parameters to monitor disease progression and immune response, highlighting the sensitivity of seahorses to pathogen invasion and the crucial role of TLR5 in mediating immune responses [194]. 

## 7. Antimicrobial Susceptibility

### 7.1. E. tarda Susceptibility Profiles: Human Infections

There has been little change in the susceptibility pattern of *E. tarda* strains recovered from human infections over the past 30 years [14]. In a limited number of in vitro studies conducted on the susceptibility of edwardsiellae recovered from different clinical sites, all studies have yielded a consensus profile for this species of universal susceptibility to most major classes of antimicrobial agents, excluding colistin and polymyxin B [195,196,197]. These results are independent of the testing method chosen. Clinical isolates of *E. tarda* are uniformly susceptible to aminoglycosides, ampicillin, ampicillin/sulbactam, β-lactam antibiotics (cephalosporins), β-lactamase-inhibitor agents, carbapenems, quinolones, aztreonam, and chloramphenicol. Such in vitro studies are supported by recent case studies [155,168,169] and one retrospective survey of *E. tarda* bacteremia in Japan [149] yielding identical results. The term “pan-susceptible” has been coined in referring to this unusual highly susceptible *Edwardsiella* species [155]. While the vast majority (>90%) of *E. tarda* strains have also been found to be susceptible to both tetracycline class compounds and trimethoprim-sulfamethoxazole, rare resistant isolates have been detected in vitro or in vivo [197,198].

### 7.2. Edwardsiella Susceptibility Profiles: Piscine Species

It is crucial to emphasize the importance of employing the correct methodology for antimicrobial susceptibility testing of *Edwardsiella* species, as outlined by the Clinical and Laboratory Standards Institute (CLSI). According to CLSI standards such as M100 [199] or VET03/04 [200], the Kirby–Bauer disk diffusion procedure is recommended for testing enterobacteria such as *Edwardsiella* species. 

In the previous report, oxytetracycline, florfenicol, and oxolinic acid have been effectively utilized against *E. tarda* in fish, with tetracycline commonly prescribed in Asia for this infection [69,201,202]. Meyer and Bullock [203] demonstrated that administering oxytetracycline in the feed (55 mg/kg for 10 days) significantly reduced mortality in catfish within three days. Similarly, an outbreak in brook trout in Quebec, Canada, was successfully managed by mixing oxytetracycline with feed and vegetable oil at 100 mg/kg live weight [182]. Additionally, *E. ictaluri* has shown susceptibility to a broad spectrum of antimicrobials, including aminoglycosides, cephalosporins, penicillins, quinolones, tetracyclines, chloramphenicol, nitrofurantoin, and potentiated sulfonamides [204]. In vitro studies further confirm that *E. piscicida* strains from various hosts and geographical locations remain susceptible to commonly used antibiotics for treating edwardsiellosis, such as enrofloxacin, oxytetracycline, trimethoprim/sulfamethoxazole, and florfenicol [69,86,112,205,206].

In the recent analysis conducted by broth microdilution methods, the *Edwardsiella* species, including *E. anguillarum*, *E. hoshinae*, *E. ictaluri*, *E. piscicida*, and *E. tarda,* have been reported that show low MIC values for amikacin, tobramycin, carbapenems (doripenem, ertapenem, imipenem, meropenem), cephalosporins (cefepime, ceftazidime, ceftriaxone, ceftiofur), quinolones (ciprofloxacin, levofloxacin, enrofloxacin), and florfenicol but high MIC values representing the decrease in susceptibility for gentamicin, streptomycin, cefazolin, macrolides (erythromycin, tylosin tartrate), penicillins (amoxicillin, ampicillin, penicillin), tetracyclines (minocycline, oxytetracycline), clindamycin, and aztreonam [112]. In another study, *E. ictaluri*, *E. hoshinae*, and *E. tarda* have been reported to have low MIC values for gentamicin, ampicillin, cefotaxime, piperacillin, imipenem, aztreonam, ciprofloxacin, and doxycycline with an antimicrobial composition below 1 µg/mL or even 0.5 µg/mL [1].

It is essential to determine the number of antimicrobial susceptibility tests conducted on *Edwardsiella* species, as evaluating the results against their epidemiological cut-off values (EC_wt_) is critical for accurate assessment of their antimicrobial susceptibility [207].

## 8. Prevention and Control

### 8.1. Breaking the Chain

*Edwardsiella* infections are already an important and major problem in the fishing industry, particularly in commercial aquaculture systems. Human infections, although still uncommon in many geographic regions of the world, are significant pathogens in Asia (e.g., Japan) and now appear to be increasingly reported in non-Asian settings. A traditional control strategy for many infectious diseases is to “break the chain” of infection-related events, that is, to eliminate one or more steps leading to ongoing cycles of infection in susceptible hosts. Unfortunately, the factors that regulate *Edwardsiella* human infections and outbreaks of disease in piscine species are quite different (hosts, frequency, disease syndromes, contact routes), although both groups are unequivocally linked together directly or indirectly via environmental (water) exposure. In a review of zoonotic bacterial infections of aquatic origin, Haenen et al. [157] list two major routes of human infections: (1) topically acquired illnesses through exposure to aquatic animals or their products and (2) consumption of raw or undercooked aquatic products. In the case of fish infections, factors controlling illnesses are much more complicated and complex as piscine species live in a variety of different marine and freshwater environments regulated by temperature, organic content, and crowding just to name a few.

Section 3 (above, Table 3) covers the main routes and reservoirs of infection for *E. tarda*-transmitted illnesses on a worldwide basis. Some of these factors require additional commentary. While it is highly unlikely that certain dietary habits traditionally associated with Southeast Asian cuisine will ever appreciably change, certain measures could be taken to reduce both morbidity and mortality. These include warning labels at the point of retail sales or at restaurants advising customers of the risk of eating raw or undercooked fish or seafood. Since some vehicles of infection, notably *Vibrio* species, play an even more important role in disease pathogenesis in this setting, such customer warnings should be mandated globally.

Pet ownership as a hobby is increasing overall. Even though traditional pets (i.e., dogs and cats) do not transmit *E. tarda*, the frequency of less common exotic animals as pets is increasing. In the US, 1.8 million households have reptiles, 1.3 million turtles, 726,000 lizards, and approximately 550,000 snakes [208]. Currently, zoonotic-related *E. tarda* illnesses have been reported on only a couple of occasions and are associated with turtles [47,55]. However, *E. tarda* has been isolated from various reptiles including snakes, iguanas, and lizards, although definitive studies are lacking [186,208,209,210,211]. Tropical or saltwater fish are maintained in aquariums in over 20 million households in the US [63,208]. These fish present potential infectious complications through traumatic events (catfish spine puncture) or direct contact of superficial sores or abraded skin with aquatic environs while cleaning tanks [63]. Recent data suggest that ornamental fish, particularly goldfish, may play a noteworthy role in *Edwardsiella* pathogenesis based on recent reports [47,56,57,58]. Finally, recent biotherapy applications such as fish pedicures or ichthyotherapy may also lead to STI as it has with other aquatic pathogens such as *Aeromonas* [212]. Recommendations for the prevention of such pet-associated infections have been published and include the suggestion that children under 5 years, immunocompromised persons, and older adults should not touch amphibians, reptiles, or their environments and hands should be washed thoroughly after handling any of these animals [213]. Gloves should be worn when cleaning aquariums [63]. All such animals and their equipment should be kept well clear of where food is prepared, consumed, or served [213].

Any decline in the number of human infections attributed to *E. tarda* will involve better education of both the public and the patient. In addition to warning signs or labels for consumable products, pet stores should have pamphlets available for customers when purchasing exotic pets (reptiles, amphibia, fish) that could trigger infectious complications when mishandled, particularly by immunocompromised persons. Comparable warnings should also be addressed by veterinarians for similar populations [214]. In healthcare settings, physicians should be alerted to possible *Edwardsiella* infections when a medical history indicates consumption of raw or undercooked fish or seafood, exposure to marine or other aquatic ecosystems, or that the patient has a professional occupation linked to these risk factors, such as fishermen, zookeepers, and dock workers (Table 3). For persons with serious underlying conditions, doctors should caution patients not to purchase or keep pets that significantly increase their risk of developing serious *Edwardsiella* disease.

*Edwardsiella*, an aquatic bacterium, adeptly transitions between free-living and host-associated lifestyles, making it a central component of the aquatic resistome. Because of the ubiquitous characteristics, to effectively break the chain of Edwardsiellosis infections, it is crucial to manage both the environment and the host interactions through environmental management, control of host interactions, sanitation and hygiene, health monitoring, nutritional support, and applications and development of vaccines and drugs [215]. 

Maintaining optimal environmental conditions in ponds is crucial to effectively prevent edwardsiellosis, prevalent across numerous farms. It is essential to regulate the physicochemical parameters of the environment to inhibit the onset of infections. Hatcheries should uphold stringent hygiene and sanitation practices. Moreover, continual monitoring for the presence of the disease and maintaining pathogen-free stocks are key strategies in controlling Edwardsiellosis effectively [67]. Changes in water quality, such as temperature, pH, dissolved oxygen, malnutrition, and overcrowding, should be mitigated to prevent outbreaks of diseases such as Edwardsiellosis. Introducing antistress agents like probiotics, ascorbic acid, and lipopolysaccharides into the feed can be beneficial. Probiotics, which consist of live or dead microbial metabolites, improve the gastrointestinal microbiota and enhance enzymatic activity, thereby boosting the host’s immune response. Studies have shown that probiotics like *Lactobacillus* spp. can enhance phagocytic activity and protect fish from acute septicemic death by boosting the alternative complement system [67,216]. 

Considering the species-specific nature of protective immunity against *Edwardsiella* infections, the efficacy of any vaccine candidate targeting this genus is contingent upon the accurate representation of the *Edwardsiella* species prevalent in the target environment. This necessitates a thorough assessment of both the local disease burden and the epidemiological prevalence of specific *Edwardsiella* species. Such evaluations are imperative to ensure that the vaccine formulation encompasses the most clinically significant strains, thereby optimizing the vaccine’s protective efficacy within aquaculture settings. This strategic approach enhances the potential for successful immunization campaigns and contributes to more sustainable disease management practices in aquaculture [69,173].

### 8.2. Vaccine Development

Recent vaccine trials in China for *Edwardsiella* species have shown significant progress in developing immunization strategies against this pathogen across various fish species. These trials have employed a range of vaccination approaches, including live attenuated vaccines, recombinant vaccines, and subunit vaccines. These vaccines were administered through different methods such as intraperitoneal injections, immersion, and a combination of immersion and oral feeding [173]. The various vaccine trials reported in the last decade, as illustrated in Table 11, have employed a range of antigen-preparation methods across different fish species, notably resulting in a diversity of relative percentage survival (RPS) values. The trials predominantly focused on olive flounder, with intraperitoneal injections being the common method of vaccine administration [77,173]. The RPS values observed ranged widely from 45% to 100%, indicating varying degrees of efficacy across different vaccine formulations and experimental conditions. These trials, conducted primarily in China and South Korea, underscore the significant effort to develop effective vaccines against *E. tarda* in aquaculture, particularly to enhance the health and survivability of economically important fish species [77,173,217,218]. 

Vaccine trials involving different vaccine types and administration methods have yielded RPS values ranging from 35.7% to 83% in turbot [77]. These methods include immersion with recombinant attenuated vaccines, intraperitoneal and intramuscular injections of live and recombinant vaccines, and combined immersion and oral feeding strategies. The administration of recombinant flagellar protein *Flg*D through intramuscular routes has consistently achieved an RPS of 70%, demonstrating the effectiveness of these vaccine approaches in zebrafish. In flounder, various vaccination strategies, including intraperitoneal injection of recombinant vaccines and a combination of oral and immersion routes, have resulted in high RPS values of up to 88.9%. This also includes the use of DNA vaccines and recombinant subunit vaccines, which have shown significant protection with RPS values typically above 60% [77]. Authors have also reported that combined immersion–oral prime-boost vaccination confers relatively good protective immunity against lethal challenges with *E. ictaluri* in Vietnamese catfish (*Pangasianodon hypophthalmus*) [77,218,219]. Furthermore, repeated oral boosting might be an effective alternative to maintain the level of immunity in Tra catfish against lethal exposure to the pathogen [219].

**Table 11:** Comprehensive summary of vaccine trials for *Edwardsiella* species.

Fish Species	Vaccine Type/Strain	Administration Route	Region	Relative Percentage Survival (RPS)	Targeted *Edwardsiella* Species
Olive Flounder	Various recombinant and live attenuated vaccines	Intraperitoneal, immersion	China, South Korea, Japan	45–100%	*E. tarda*
Turbot	Recombinant attenuated and others	Immersion, intraperitoneal	China	35.7–83%	*E. tarda*
Zebrafish	Recombinant flagellar protein FlgD	Not specified	China	70%	*E. tarda*
Flounder	Recombinant, subunit, DNA vaccines	Intraperitoneal, oral, immersion	China	60–88.9%	*E. tarda*
Rohu	Attenuated *E. tarda* strain	Immersion	India	80%	*E. tarda*
European Eel	Recombinant protein vaccines	Intraperitoneal	Spain	75–85%	*E. tarda*
Large Yellow Croaker	Live attenuated vaccine	Oral	China	85.7%	*E. tarda*
Channel Catfish	Inactivated vaccine	Immersion	China	62%	*E. tarda*
Japanese Flounder	DNA vaccine	Injection	China	85%	*E. tarda*
Catfish	Inactivated *E. ictaluri* (outer membrane proteins)	Intraperitoneal (IP)	-	-	E. ictaluri *

References [77,173,218,220]. * The vaccine was licensed for Vietnam by AQUAVAC^®^ (AQUAVAC-ESC, https://www.aquavac-vaccines.com/ accessed on 12 May 2024) and Pharmaq^®^ (ALPHA JECT^®^ Panga 2, https://www.pharmaq.no/ accessed on 12 May 2024).

## 9. Conclusions

While much progress has been made in the study of *Edwardsiella* and edwardsiellosis in relation to both human and animal (e.g., fish) infections, much work remains to be accomplished. Recent taxonomic investigations leading to the reidentification of “*E. tarda*” isolates from both humans and animals, accompanied by the description of new species, strongly suggest that new species-associated environmental distributions, disease spectrums, virulence characteristics, and other phenotypic properties may be uncovered. Unfortunately, little work has been published since the 1970s on the frequency and distribution of edwardsiellae in various reptiles and animals other than fish. To fully elucidate the transmission dynamics of *Edwardsiella* among animals, it is essential to conduct diverse prevalence studies. Additionally, the adoption of epidemiological cut-off values as a novel method for assessing antimicrobial susceptibility is imperative. This approach should be extended to pathogens within the *Edwardsiella* genus to facilitate the development of globally applicable, species-specific antimicrobial treatment protocols. Furthermore, although many potential virulence genes or characteristics have been identified on both a phenotypic and molecular basis, we still have a relatively poor understanding of how such toxins or factors might be operative in vivo. Finally, better recognition and education by the private, public, and professional sectors concerning *Edwardsiella* and related risk factors needs to be undertaken to prevent, control, and contain extraintestinal illnesses and outbreaks through the use of vaccines or other preventive measures.

## Figures and Tables

**Table 1:** Seminal events in the early history of the genus *Edwardsiella* and *E. tarda.*

Years	Event	Comment	Reference
1959–1962	Organisms first recovered and reported by Sakazaki and Tamura	“Asakusa group”; 153 isolates with similar characteristics	[2]
1962	Hoshina describes *Paracolobactrum anguillimortiferum*	Apparently an *Edwardsiella* but extant cultures are not available; no type strain deposited	[4]
1964	Report on “unidentified” group in family *Enterobacteriaceae*	Labeled the “Bartholomew” group	[3]
1965	New genus and species	Formal description of *Edwardsiella* and *E. tarda*; covered 37 different strains	[5]
1967	Detailed description of *E. tarda* strains	“Asakusa group”, 248 cultures; CDC *E. tarda* strains, 225 cultures	[2,10]
1968	First report of invasive disease caused by *E. tarda*	Meningitis in immunocompromised person	[8]
1971	Association with gastroenteritis in Thailanders	Of 23 persons (ages 8 mos–80 years;) with stool culture or rectal swab, 18 (78%) symptomatic, 4 asymptomatic and healthy, 1 with *Shigella* dysentery	[11]

**Table 2:** Current species in the genus *Edwardsiella.*

Species	Type Strain	Nomenclature ^a^		Major Source(s) ^b^	Pathogenic for:		Ref.
		Validated	Correct		Humans	Fish	
*E. anguillimortifera*	ATCC 15947^T^	Yes	No	Snakes			[19]
*E. tarda*	ATCC 15947^T^	Yes	Yes	Human	+	±	[5]
*E. hoshinae*	ATCC 33379^T^ (=CIP 78.56)	Yes	Yes	Birds, Reptiles	-	-	[19]
*E. ictaluri*	ATCC 33202^T^	Yes	Yes	Catfish	-	+	[20]
*E. piscicida*	CCUG 62929^T^ (=NCIMB 14824^T^)	Yes	Yes	Fish	-	+	[21]
*E. anguillarum*	CCUG 64215^T^	Yes	Yes	Eels	-	±	[22]

^a^ List of prokaryotic names with standing in nomenclature [24]. ^b^ from species proposal and description.

**Table 3:** Documented and suspected sources and vehicles of *Edwardsiella* transmission.

Category	Examples
Animal Exposure	Turtle, Ornamental fish
Aquatic Exposure	Bathing in village pond, Diving, Fall in brackish water, Fall in canal, Freshwater lakes, Immersion in lake (baptism), Near-drowning, Washed clothes in river, Swimming
Food Consumption	Ayu, Catfish, Ceviche, Eel, Fish (unspecified), Flounder, Horse mackerel, Meat (raw), Oysters, Sashimi, Seafood soup, Shark meat, Shrimp, Sushi, Tuna
Occupation/Vocation	Caregiver, Crabbing, Dock maintenance worker, Farmers, Fisherman, Fishmonger, Gardeners, Hobbyists, Veterinarians, Zoo staff
Trauma	Automobile accident, Brick, Catfish spine, Fishbone, Glass

References: [45,46,47,48,49,50,51,52,53,54,55].

**Table 4:** Goldfish-associated *E. tarda* infections.

	Spencer et al. [57]	Hasegawa et al. [47]	Gilani et al. [56]	Tsuchiya et al. [58]
Age/Sex	8/M	25/F	4/F	77/M
Comorbid conditions	Renal transplant	Sigmoid sinus thrombosis	None	Thoracic spondylitis, diabetes, prostate and pancreatic cancer
Risk factors/Mode of transmission	Playing in aquarium water with pet goldfish	Had a goldfish and a turtle	Goldfish in tank (died); hand in aquarium multiple times	Taking care of goldfish before admission
Symptoms	Weight loss, abdominal cramping, bloody stools	Intrauterine infection	Fever, dysuria	Back pain
Duration	3 weeks	Not available	1 week	Several weeks
Diagnosis	Gastroenteritis	Bacteremia	Urinary tract infection	Thoracic spondylitis Sepsis
Positive culture(s)	Stool	Blood	Urine (>10^5^ CFU)	Blood, urine, abscess
Concurrent organisms	None	None	None	None
Outcome	Resolved	Resolved	Resolved	Resolved

**Table 5:** Defining biochemical traits for inclusion in the family *Enterobacteriaceae.*

Character	*Enterobacteriaceae*	The Genus *Edwardsiella*	Exceptions
Gram-negative rod	+	+	None
Facultative metabolism	+	+	None
Possession of the ECA ^a^	+	+	None
Spore formation	-	-	None
Cytochrome oxidase	-	-	None
Catalase	+	+	None
Nitrate reductase	+	+	None
Fermentation of D-glucose	+	+	*E. ictaluri*
Fermentation of D-xylose	+	-	None

References: [1,14,19,20,44,102,103]. ^a^ ECA, enterobacterial common antigen.

**Table 6:** Biogroups of *E. tarda.*

Property	Biotypes		
	Wild Type	Biogroup 1	“Biogroup 2”
Indole production	+	+	+
H_2_S production	+	- ^a^	- ^a^
Fermentation of:			
D-mannitol	-	+	-
L-arabinose	-	+	-
Sucrose	+	+	+
Tetrathionate reduction	+	-	ND
Present in human clinical specimens	+	+ ^b^	+
Human pathogen	+	(+) ^b^	+

ND, not determined. ^a^ method dependent; ^b^ based upon data from Grimont et al. [19].

**Table 7:** Biochemical differences between *E. ictaluri*, *E. piscicida*, and *E. tarda.*

Property	Species		
	*E. ictaluri*	*E. piscicida*	*E. tarda*
Indole production	-	+	+
H_2_S production	-	^+^	+
Motility (25 °C)	-	+	+
Motility (37 °C)	-	+	+
Growth (37 °C)	-	+	+
Growth (42 °C)	-	-	+
RBC hemolysis	alpha	beta	beta
Methyl red	-	+	+
Malonate	-	-	-
Fermentation of:			
Sucrose	-	-	-
Trehalose	-	-	-
D-mannitol	-	v	v
Arabinose	-	v	v

v, variable.

**Table 9:** Edwardsiellosis outbreaks in fish *.

Agent	Mortality Rate	Fish Species	Water Temperature	Year	Region/Ref.
*E. ictaluri*	40–50%	Hybrid red tilapia juveniles	25–30 °C	2016	Northern Vietnam [84]
*E. piscicida* and *E. tarda*	~1.0%	Barramundi	~28 °C	2016–2017	Michigan, US [76]
*E. tarda*	5%	Korean catfish	24–26 °C	2009	Korea [179]
*E. tarda*	5–15%	Fourfinger threadfin	ND		Taiwan [181]
*E. piscicida-like*	20%	White grouper	ND	2011–2012	Israel [124]
*E. tarda*	30%	Brook trout	18–19 °C	1998	Canada [182]
*E. tarda*	3–10%	Turbot	15.2–17.7 °C	2003	Atlantic Coast of Spain [183]
*E. tarda*	80%	Japanese flounder	20 °C	1985	Hokkaido [184]
*E. anguillarum*	20%	Nile tilapia	30 °C	2019	Chungbuk Province of Korea [87]

*: Only reports were presented that included mortality rate; ND: no data.

**Table 10:** The most commonly reported virulence genes in *Edwardsiella* species.

Virulence Gene	Detected in *Edwardsiella* Species	Mechanism
*hem*X, *hem*C, *hem*D, *hem*N, *hem*M, *hem*S, *hmu*T	*E. tarda*	Involved in heme biosynthesis and iron utilization, crucial for bacterial virulence and survival in host environments
*fur*	*E. tarda*	Ferric uptake regulator, controls iron metabolism and is linked to the expression of other virulence genes
*bas*S	*E. tarda*	Sensor protein involved in regulation and cell signaling, possibly related to pathogen virulence
*flh*B, *flh*A, *mot*A, *fli*G, *fli*R	*E. tarda*	Related to flagellar biosynthesis and motility, important for bacterial movement and host tissue colonization
*tol*C, *tol*B	*E. tarda*	Involved in protein translocation and drug efflux, contributing to bacterial resistance and pathogenicity
*mlt*C, *yjf*G, *imp*, *mrc*B	*E. tarda*	Related to cell wall and capsule formation, crucial for structural integrity and evasion of host immune defenses
*omp*W	*E. tarda*	Outer membrane protein that may be involved in interactions with the host’s immune system
*pst*C, *pst*B, *pst*S	*E. piscicida*	Phosphate transport system components, important for bacterial metabolism and survival in the host
*isor*	*E. piscicida*	Iron sulfate oxidoreductase, plays a role in iron metabolism essential for pathogenicity
*gad*B	*E. piscicida*	Glutamate decarboxylase, may contribute to acid resistance and virulence
*kat*B	*E. piscicida*	Catalase, involved in combating oxidative stress within the host
*fim*A	*E. piscicida*, *E. tarda*	Fimbrial protein important for adhesion to host tissues
*qse*B, *qse*C	*E. tarda*	Two-component regulatory system influencing virulence and possibly quorum sensing
*tna*A	*E. tarda*	Tryptophanase, involved in indole production and potentially modulating host immune responses
*dna*J, *htp*G	*E. tarda*	Heat shock proteins, implicated in stress response and possibly in virulence modulation

References [16,33,41,43,67,77,190].

## Data Availability

No new data were created or analyzed in this study. Data sharing is not applicable to this article.
